# Measles-based Zika vaccine induces long-term immunity and requires NS1 antibodies to protect the female reproductive tract

**DOI:** 10.1038/s41541-022-00464-2

**Published:** 2022-04-19

**Authors:** Drishya Kurup, Christoph Wirblich, Rachael Lambert, Leila Zabihi Diba, Benjamin E. Leiby, Matthias J. Schnell

**Affiliations:** 1 Department of Microbiology and Immunology, Sidney Kimmel Medical College at Thomas Jefferson University, Philadelphia, PA 19107 USA; 2 Division of Biostatistics, Department of Pharmacology and Experimental Therapeutics, Sidney Kimmel Medical College, Thomas Jefferson University, Philadelphia, PA 19107 USA; 3 Jefferson Vaccine Center, Sidney Kimmel Medical College, Thomas Jefferson University, Philadelphia, PA 19107 USA

**Keywords:** Live attenuated vaccines, Viral infection

## Abstract

Zika virus (ZIKV) can cause devastating effects in the unborn fetus of pregnant women. To develop a candidate vaccine that can protect human fetuses, we generated a panel of live measles vaccine (MV) vectors expressing ZIKV-E and -NS1. Our MV-based ZIKV-E vaccine, MV-E2, protected mice from the non-lethal Zika Asian strain (PRVABC59) and the lethal African strain (MR766) challenge. Despite 100% survival of the MV-E2 mice, however, complete viral clearance was not achieved in the brain and reproductive tract of the lethally challenged mice. We then tested MV-based vaccines that expressed E and NS1 together or separately in two different vaccines. We observed complete clearance of ZIKV from the female reproductive tract and complete fetal protection in the lethal African challenge model in animals that received the dual antigen vaccines. Additionally, MV-E2 and MV-NS1, when administered together, induced durable plasma cell responses. Our findings suggest that NS1 antibodies are required to enhance the protection of ZIKV-E antibodies in the female reproductive tract.

## Introduction

Zika virus (ZIKV) is an emerging mosquito-borne pathogen. In most healthy adults ZIKV causes only mild infection, but in rare cases, causes Guillain-Barré syndrome (GBS) in adults and congenital Zika syndrome (CZS) in infants born to ZIKV-infected mothers^1^. ZIKV is primarily transmitted by mosquitoes of the *Aedes* genus but can also be transmitted through congenital, perinatal, blood transfusion, and sexual routes^2^. Before the 2007 ZIKV outbreak in the Yap Islands, only 14 human cases were reported worldwide (WHO, Accessed July 29, 2021). Since then, outbreaks have occurred in the Pacific islands, South and Central America, and the Caribbean (WHO, July 29, 2021). These unprecedented outbreaks led to a sudden increase in human cases with several symptomatic infections characterized by fever, conjunctivitis, rash, headache, myalgia, and arthralgia^3^. Also, retrospective studies of the epidemic showed a strong correlation of ZIKV disease with microcephaly and/or other congenital disabilities in infants and GBS in adults^4^. Based on these case studies, WHO declared ZIKV a Public Health Emergency of International Concern on Feb 1, 2016 (WHO, Accessed July 29, 2021). Recently, in July 2021, Zika virus infections were reported in India, suggesting that this virus may reemerge, causing a public health concern^5^.

ZIKV belongs to the *Flavivirus* genus of the *Flaviviridae* family. ZIKV contains a single-stranded positive-sense RNA genome containing a 5′ untranslated region (UTR), a single open reading frame (ORF) encoding a polyprotein, and a 3′ UTR. The ORF encodes three structural proteins (capsid, C; pre-membrane, prM; and envelope, E) and seven nonstructural proteins (NS1, NS2A, NS2B, NS3, NS4A, NS4B, NS5)^6^. The prM protein associates with E to form heterodimers and is essential for the proper folding of E^7^. Among the structural proteins, the E protein is the major virion surface protein, but the M protein is also displayed on the surface of the viral particle. C protein is a major internal protein surrounded by the host-derived spherical lipid bilayer membrane^8^. The glycosylated NS1 protein forms a homodimer and separates into three distinct populations: a large portion localizes to the site of viral RNA synthesis and is critical for replication; a second minor portion traffics to the plasma membrane (mNS1, membrane) where it forms a hydrophobic spike, which may contribute to its cellular membrane association; and a third is secreted into the extracellular space as a hexamer (sNS1, secreted)^9^. The sNS1 is secreted in the serum of infected individuals in high concentrations and is used as a diagnostic biomarker^10^. There are two distinct strains of ZIKV, the Asian and African, but only one serotype^11^. The Asian strain associated with the recent outbreaks evolved from the first isolated 1947 African strain after sporadic cases of ZIKV in Africa and Asia (WHO, November 12, 2019).

ZIKV infection in healthy adults generates virus-neutralizing antibodies (VNAs) directed towards the E protein and antibodies directed towards the NS1 protein. The presence of ZIKV-E and NS1 antibodies are suggestive of protective immunity in humans^12^. While the protective E protein antibodies neutralize and target the virions, the NS1 antibodies are non-neutralizing and may target the infected cells or secreted form of NS1, thereby blocking its potential effects on immune evasion and pathogenesis^13^. Several candidate vaccines have utilized the ZIKV ME, prME, and/or NS1 as the immunogen of choice in DNA, RNA, viral vectors, live attenuated vaccine (LAV), inactivated virus, and subunit vaccine platforms. Some of these vaccines have proceeded to Phase I/II clinical trials that demonstrated safety and immunogenicity^14–20^. When these vaccine platforms were tested in non-pregnant mice and monkeys, they achieved systemic viral clearance. However, the vaccines tested in pregnant mouse and monkey models achieved incomplete protection in fetuses^21,22^. The RhAd52- and Ad26-based ZIKV vaccine was recently tested in pregnant *IFNαβR*^*−/−*^ mice were more successful: just marginal levels of ZIKV RNA were detected in the placenta and fetal brains^23^.

Each of these vaccine platforms has its advantages and shortcomings. The DNA, RNA, VLP, and subunit vaccine are likely safe platforms that require multiple doses, but the longevity of the vaccine-induced immune responses in humans is unknown. Viral vectors, like modified vaccinia virus Ankara (MVA) and vesicular stomatitis virus (VSV), induce durable responses but have safety implications when administered to children < 1 year of age, pregnant women, and the immunocompromised^24,25^. While protective parameters are yet to be established for ZIKV, the development of a certain threshold of neutralizing E protein antibodies is considered protective for other flaviviruses, such as yellow fever (YF), tick-borne encephalitis virus (TBEV), and Japanese encephalitis virus (JEV). But ZIKV, unlike other flaviviruses, can cause devastating effects in pregnant women, resulting in prolonged viremia leading to CZS in their infant^26^. Hence a ZIKV vaccine must prevent viremia in pregnant women and their fetuses.

To safely and effectively overcome the persistence of viremia in pregnant women and the impact on their fetuses, we developed a ZIKV vaccine based on the measles vaccine vector. The measles virus (MV) vaccine has more than 50 years of historical data confirming its safety and long-term efficacy by induction of durable neutralizing antibody and T cell-mediated immunity^27^. Children <1 year of age, pregnant women, and postpartum women can be MV vaccinated^27^. The success of the MV vaccine has led to its development as a vaccine vector for DENV, ZIKV, WNV, HIV, Middle East respiratory syndrome (MERS), and malaria antigen^27^. The first measles based Zika vaccine was generated by Nürnberger et al. wherein they developed a Schwarz strain MV that encodes ZIKV prM and soluble E proteins (without the stem and transmembrane domain of E) and observed complete protection of fetuses and reduced viral loads in the placenta of vaccinated IFNAR^−/−^ -CD46 mice when challenged with a ZIKV Asian strain (French Polynesian).

In this study, we used an MV vaccine vector (Edmonston B strain) to generate a candidate ZIKV vaccine included in the childhood vaccination regimen. We inserted the codon-optimized (co) ZIKV prME from the Asian PRVABC59 strain into the transcription cassette before N (position 0) and another between N and P (position 2), generating the MV-E(0) and the MV-E(2) vaccine, respectively. Both vaccines completely cleared the virus in mice challenged with the non-lethal ZIKV PRVABC59 (Asian) 2015 strain and dramatically reduced ZIKV RNA copies when challenged with the lethal highly neurotropic mouse-adapted ZIKV MR766 (African) strain. Second-generation vaccine constructs containing the ZIKV NS1 protein-MV-NS1(2) were tested singly or in combination with MV-E(2) or by inserting the co ZIKV prMENS1 between H and L protein (position 6, MV-E-NS1(6)) to allow for enhanced protection using the lethal mouse-adapted ZIKV African MR766 strain. Our results showed that NS1 antibodies alone did not protect as the MV-NS1(2) mice showed signs of ZIKV disease when challenged with a lethal Zika African strain. Interestingly, the combination vaccine, MV-NS1(2) and MV-E2 virus and the MV-E-NS1(6) vaccine provided better protection than MV-E2 alone in terms of clearing ZIKV RNA in the female reproductive tract and fetuses. Lastly, the combination vaccine induced ZIKV-E-, ZIKV-NS1-, and MV-H-specific plasma cell responses. These findings suggest that further development of a dual antigen vaccine could lead to an effective pre-exposure Zika vaccine for children.

## Results

### Design, recovery, and characterization of first-generation MV-ZIKV vaccines

**Fig. 1:**
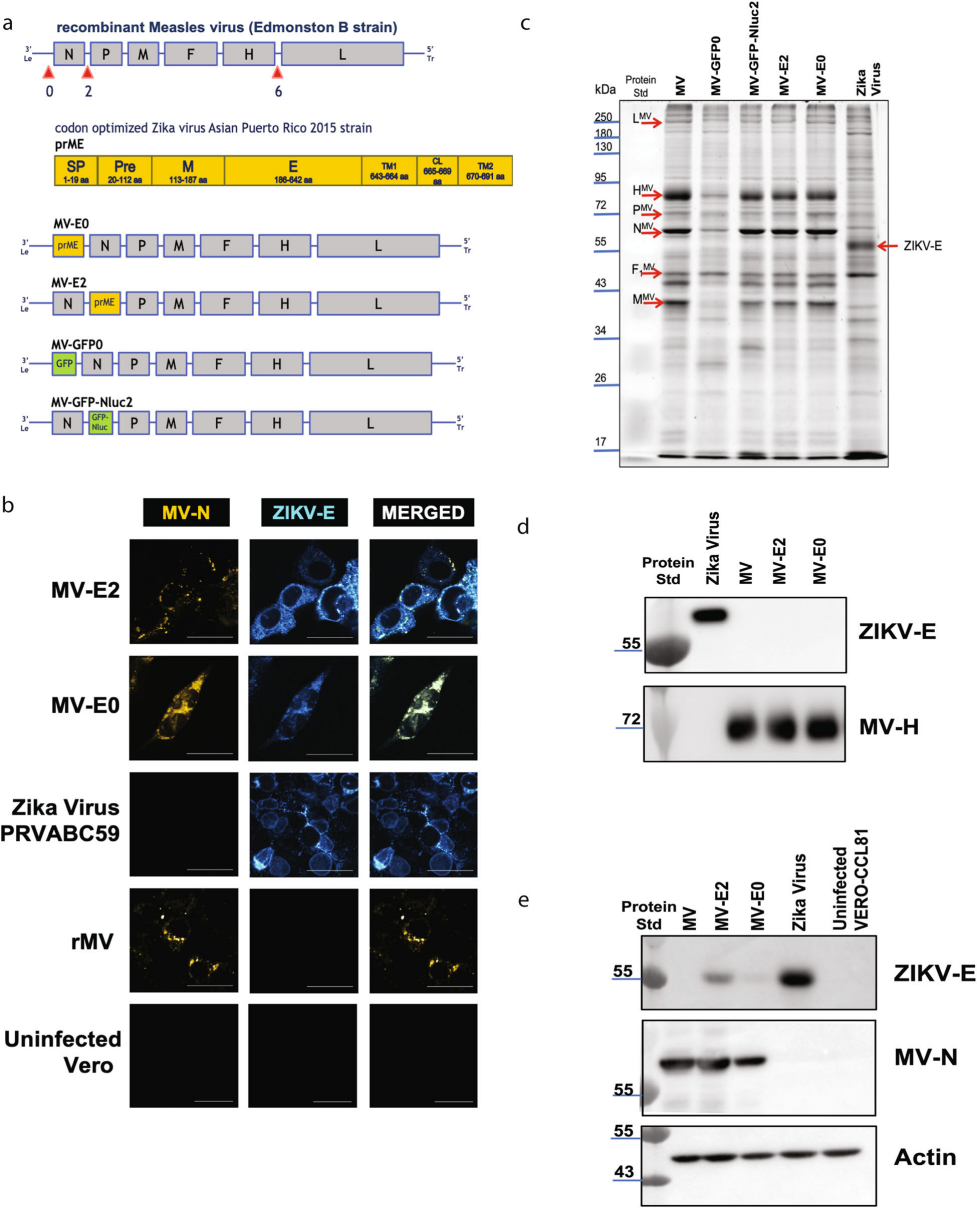
Generation and characterization of first-generation MV-based candidate ZIKV vaccines. **a** MV-ZIKV vaccine constructs and controls. **b** Immunofluorescence staining of Vero cells were infected at MOI-0.1 for 72 h with the recovered MV-ZIKV candidate vaccines and control viruses. The permeabilized cells were stained for MV using an α-MV nucleoprotein mouse monoclonal and for the ZIKV-E using a mouse monoclonal antibody (Biofront 1176-56). The MV-N cl25 antibody (Sigma, NP cl.25) is conjugated with a Dylight 488 (green color changed to Yellow for visualization) fluorophore and ZIKV-E is stained with a secondary goat α-mouse Cy-3 (Jackson Immunoresearch, red color changed to Cyan for visualization) antibody. Confocal images were taken using NIKON-A1R, 60X magnification, 3X zoom. The scale bar measures 30 μm. The data is representing one replicate of *n* = 5. **c** Sucrose-purified virions were analyzed on SDS-PAGE (10%) stained with SYPRO Ruby. Zika virus PRVABC59 strain was loaded as the control. The data is representing one replicate of *n* = 4. **d** Western blot analysis of sucrose-purified virions probed for ZIKV-E (Biofront mouse mAb) and MV-H (Rabbit polyclonal sera). Zika virus PRVABC59 strain was loaded as the control. The data is representing one replicate of *n* = 4. **e** Western blot analysis of cell lysates of VERO cells infected with MV-ZIKV candidate vaccine at MOI-5 for 60 h. Zika virus PRVABC59 infected cell lysates were loaded as the control. The blot was probed for ZIKV-E (Biofront mouse mAb) and MV-N (Sigma, NP-Cl25). The data is representing one replicate of *n* = 2.

Recombinant measles viruses (rMV) have been used as vaccine vectors for different infectious diseases and can serve as an excellent platform to generate an early childhood vaccine for ZIKV. A full-length measles virus cDNA clone (MV-ATS-0) that allows insertion of foreign genes upstream of the nucleoprotein gene served as the backbone of our vaccine constructs. We modified the cDNA clone by adding a hammerhead ribozyme before the leader region. We generated two additional vectors, MV-ATS-2 and MV-ATS-6, that allow insertion of foreign genes between the N and P genes and the H and L genes, respectively. We chose to evaluate multiple vectors because transcription of the measles virus genome occurs sequentially, which results in a transcription gradient as the polymerase proceeds from one gene to the next. The point of insertion also affects the replication and spread of recombinant viruses. We first inserted the codon-optimized (co) gene of the ZIKV precursor (pr), membrane (M), and envelope (E) protein (strain PRVABC59 Asian 2015) upstream (ATS-0) and downstream (ATS-2) of the nucleoprotein gene (Fig. 1a). The recombinant viruses rMV-E0 and rMV-E2 were recovered as described in the Methods section and amplified on VERO cells. Control viruses that express a green fluorescent protein (GFP) at ATS-0 and a GFP-nanoluciferase fusion gene at ATS-2 were generated similarly and used as control vaccines (Fig. 1a).

An immunofluorescence assay performed characterization of the rMV-ZIKV viruses to examine ZIKV-E and MV nucleoprotein (N) expression (Fig. 1b). Vero cells infected at an MOI of 0.1 for three days were permeabilized and stained with antibodies directed against ZIKV-E and MV-N. Co-expression of MV-N and ZIKV-E was detected for the MV-E0, and MV-E2 viruses, with only ZIKV-E, expressed in the ZIKV PRVABC59-infected cells. MV-N but not ZIKV-E was detected in the empty vector rMV infected cells.

Next, we purified the rMV-ZIKV viruses over a 20% sucrose-cushion and analyzed them by SDS-PAGE (10%). The Sypro-ruby stained gel showed the presence of all six MV structural proteins in the MV-E0 and MV-E2 viruses migrating at a similar size compared to the empty vectors rMV, MV-GFP0, and MV-GFP-Nluc2 viruses (Fig. 1c). Western blot analysis of a similar SDS-PAGE of purified rMV-ZIKV vaccines probed for ZIKV-E showed the absence of ZIKV-E in the virion (Fig. 1d), indicating that rMV-ZIKV vaccines do not incorporate the ZIKV E protein, thereby should retain the tropism of the MV vector. Western blot analysis of purified rMV-ZIKV vaccines probed for MV-H showed similar levels in the MV constructs (Fig. 1d).

Cell lysates obtained from Vero cells infected by rMV-ZIKV vaccines at an MOI of 5 for 60 h were analyzed on a western blot. The western blot probed for ZIKV-E & MV-N confirmed their expression (Fig. 1e) in both the MV-E0 and MV-E2 virus-infected cells, with lower ZIKV-E expressed by the MV-E0 virus. The lower levels of ZIKV-E expression in MV-E0 may be due to its slower replication than MV-E2 (Supplementary Fig. 4). Similar levels of the actin control were detected in the western blot of the cell lysates (Fig. 1e).

### Efficacy of MV-ZIKV vaccines using non-lethal ZIKV Asian PRVABC59 strain

The rMV vaccine strain requires both the presence of the human CD46 receptor (hCD46) for its entry into cells^28^ and the lack of the interferon αβ receptor (*IFNαβR*^*−/−*^) for its systemic replication in mice^29^. Therefore, rMV vaccines historically have been tested in *hCD46 IFNαβR*^−/−^ transgenic mice. To initially test the immunogenicity of our new vaccines, two groups consisting of five female *hCD46 IFNαβR*^*−/−*^ mice (10- to 12-weeks old) each were immunized with either MV-E2 or MV-E0 on day 0 and boosted on day 28 with 10^5^ TCID_50_ intraperitoneally (i.p.); two other groups were mock immunized with PBS at the same time points (Supplementary Fig. 1a). The mice were bled on days 0, 28, 35, and 63 and tested for the presence of ZIKV-E and MV-H IgG antibodies by ELISA.

After the boost, the MV-E2 vaccinated mice showed significantly higher titers against ZIKV-E (*p* = 0.002) than the MV-E0 vaccinated mice (Supplementary Fig. 1b). By day 63, the MV-E0 and MV-E2 vaccinated mice had similar ZIKV-E EC_50_ IgG titers (Supplementary Fig. 1b). The MV-H responses were similar for the MV-E0 and the MV-E2 vaccinated animals for all time points (Supplementary Fig. 1c). Since the vaccines showed strong immunogenicity, the mice were challenged with the 10^6^ FFU of non-lethal ZIKV Asian PRVABC59 strain subcutaneously (s.c.) on day 63 to mimic the natural infection route; they were then humanely euthanized at day 77 (14 days post-challenge). One PBS group was mock challenged with PBS and served as a control.

The MV-E2 and MV-E0 vaccines provide robust protection with undetectable ZIKV RNA in the blood of vaccinated animals. At the same time, the control PBS group had significantly high RNA copies ~10^4^- 10^7^ in the blood on days 7 and 14 post-challenge (Supplementary Fig. 1e). Similar results were observed in the brain (Supplementary Fig. 1f) and the reproductive tract (Supplementary Fig. 1g). The high ZIKV neutralizing titers seen before the challenge (Supplementary Fig. 1d) were maintained at the necropsy time point (Supplementary Figure 1h) for the MV-E2 and the MV-E0 groups.

### Efficacy of MV-ZIKV vaccines using lethal mouse-adapted ZIKV African MR766 strain

**Fig. 2:**
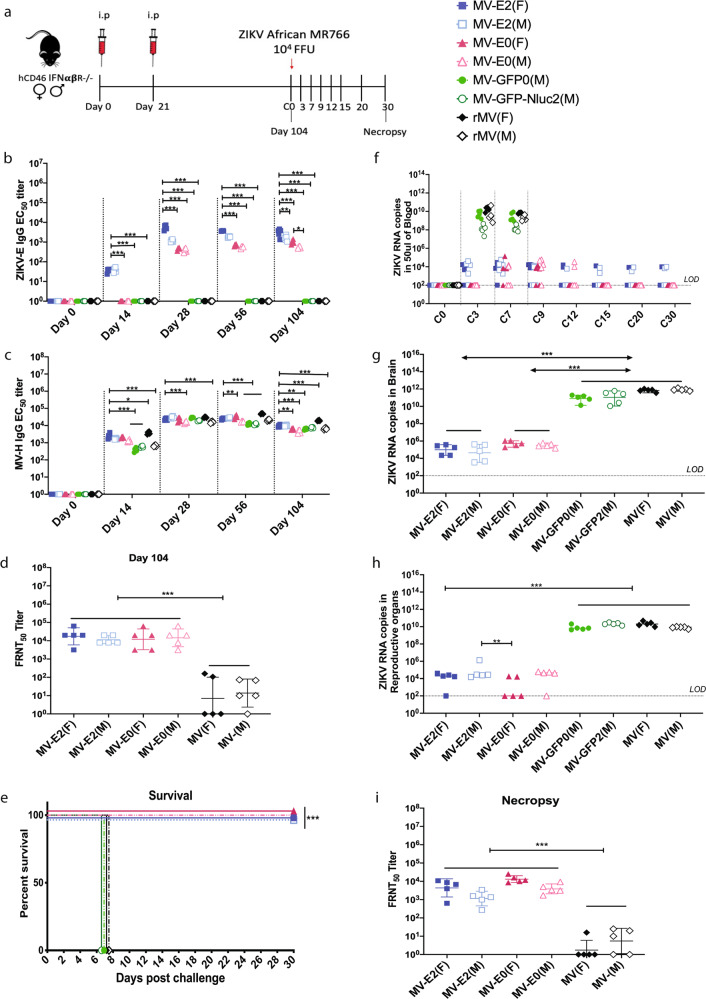
Immunogenicity and efficacy testing of first-generation candidate MV-ZIKV vaccines using a lethal ZIKV African MR766 challenge strain. **a** Timeline of vaccination, challenge and viral load determinations. Anti-ZIKV-E-specific (**b**), and anti-MV-H-specific (**c**) ELISA IgG EC_50_ titers of the vaccinated animals are plotted on a graph for all animals at different time points. The mean of triplicate EC_50_ values is depicted per animal. Mean ± SD is depicted per group. ZIKV neutralization with PRVABC59 Asian strain. FRNT was performed on day 104 (**d**) and necropsy (**i**) sera from vaccinated animals and controls. The mean 50% neutralizing titer (FRNT_50_) of triplicates is plotted for each animal on the graph. The Mean ± SD is depicted per group. **e** Kaplan–Meier survival curve analysis of vaccinated and control animals post-challenge. ZIKV RNA copies by quantitative polymerase chain reaction (qPCR) in the blood (**f**), brain (**g**), and reproductive tract (**h**). The mean of triplicate RNA copies is depicted per animal. The Mean ± SD is depicted per group. The LOD is 100 copies. Statistics for Fig. 2b–d & f–i were done using the two-way ANOVA with post hoc Tukey HSD test and performed on log-transformed data for each time point. Figure 2e survival curves were analyzed using the log-rank test with a Bonferroni correction. Only significant differences are depicted. *P* value of 0.1234(ns), 0.0332(*), 0.0021(**), 0.0002(***), <0.0001(****) are depicted accordingly. LOD stands for limit of detection. A horizontal line (—) is used to include all groups below it.

To test whether the MV-ZIKV vaccines could be efficacious in a lethal mouse challenge model in both males and females, eight groups of five female (F) or male (M) *hCD46 IFNαβR*^−/−^ mice (7- to 8-weeks old) each were immunized with 10^5^ TCID_50_ i.p. of either MV-E2(F), MV-E2(M), MV-E0(F), MV-E0(M), MV-GFP0(M), MV-GFP-Nluc2(M), rMV(F), or rMV(M) vaccine, on day 0 and boosted on day 21 (Fig. 2a). The mice were bled on days 0, 14, 28, 56, and 104 and tested for the presence of ZIKV-E and MV-H IgG antibody titers.

The ZIKV-E immune responses developed as early as day 14 in the MV-E2 group, while for the MV-E0 group, detectable responses were seen only after the boost on day 28. The MV-E2 vaccinated mice developed significantly higher ZIKV-E-specific antibody titers than the MV-E0 vaccinated animals at all time points. This difference was only observed in younger mice. Significantly higher ZIKV-E IgG antibody titers were observed in females than males in MV-E2, and MV-E0 vaccinated groups (Fig. 2b). The MV-H IgG responses were the highest in the rMV(F) at all time points, with higher MV-H antibody titers seen in the females than males in the empty MV vector group. The MV-E2 group developed significantly higher MV-H IgG antibody titers than the MV-E0, MV-GFP0, and the MV-GFP-Nluc2, indicating that the vector immunity was unaffected by the addition of the ZIKV-E into the genome (Fig. 2c).

The vaccinated animals were challenged on day 104 with a lethal dose of 10^4^ FFU s.c. of the lethal ZIKV African MR766 strain. All of the MV-E2 and MV-E0 vaccinated male and female mice survived the challenge, showing no signs of ZIKV disease, while the MV-GFP0(M), MV-GFP-Nluc2(M), and rMV(F&M) controls developed ZIKV disease by day 7 and were humanely euthanized (Fig. 2e). Significantly lower ZIKV RNA copies were observed in the blood of the MV-E2 and MV-E0 groups than the control groups (MV-GFP0, MV-GFP-Nluc2, and rMV) at all time points, with complete viral clearance from the blood seen in the MV-E0, vaccinated animals by day 15 (Fig. 2f). Similar to the viral load in the blood, significantly lower ZIKV RNA copies were observed in the brain (Fig. 2g) and the reproductive tract (Fig. 2h) of the MV-E2 and the MV-E0 vaccinated animals than the control groups. The MV-E2 and MV-E0 vaccinated animals developed similar ZIKV neutralizing titers at day 104 and necropsy (Fig. 2d and i).

### MV-ZIKV vaccines induce long-term immunity and protection

We next assessed the longevity of the immune responses induced by MV-E2 and MV-E0 vaccines. Four groups of five female *hCD46 IFNαβR*^*−/−*^ mice (9- to 12-weeks old) were vaccinated on day 0 and boosted on day 28 with 10^5^ TCID_50_ i.p. of MV-E2, MV-E0, rMV, or PBS (Supplementary Fig. 2a). The MV-E2 and MV-E0 groups had similar ZIKV-E IgG antibody titers by day 110 (Supplementary Fig. 2b). Similar to the previous experiment, MV-H IgG antibody titers were similar in the MV-E2-, MV-E0-, and the MV-vector vaccinated animals (Supplementary Fig. 2c). Of note, MV-E2 and MV-E0 vaccinated animals survived 10^4^ FFU of lethal ZIKV African MR766 strain challenge (Supplementary Fig. 2e) with viral clearance in the blood at necropsy (Supplementary Fig. 2f) and significantly lower ZIKV RNA copies in the brain (Supplementary Fig. 2g) and the reproductive tract compared to the control animals (Supplementary Fig. 2h). Reduced viral presence correlated with high ZIKV neutralizing titers on day 144 (Supplementary Fig. 2d) and necropsy (Supplementary Fig. 2i).

### MV-E2 vaccine is efficacious when administered intramuscularly

To learn whether the route of immunization is important and whether prior MV immunity affected the efficacy of the MV-E2 and MV-E0 vaccines, we performed an additional mouse challenge study. Four groups of five male or female *hCD46 IFNαβR*^−/−^ mice (9- to 12-weeks old) were pre-vaccinated (Prevac) on day –35 with 10^5^ TCID_50_ intramuscularly (i.m.) of rMV. Then, on day 0, they were vaccinated, i.m. with 10^5^ TCID_50_ of either MV-E2 or MV-E0 vaccines, and boosted on day 21 (Supplementary Fig. 3a). In addition, on day 0, seven groups of five female or male mice were vaccinated, i.m. with MV-E2, MV-E0, MV, or PBS. The ZIKV-E IgG titers followed a similar trend to the i.p. MV-E2 and MV-E0 vaccinated animals, but lower titers were seen in the i.m. vaccinated animals (Supplementary Fig. 3b). For the Prevac groups, MV-E2 vaccinated animals elicited significantly low ZIKV-E IgG titers, while the MV-E0 vaccinated animals did not seroconvert throughout the study (Supplementary Fig. 3b). The MV-H IgG titers were boosted on days 0 and 21 in the Prevac groups, which indicated successful vaccination. The Prevac groups developed similar MV-H antibody responses by day 56 to the MV-E2, MV-E0, and rMV groups (Supplementary Fig. 3c). All the animals were challenged on day 63 with 10^4^ FFU of lethal ZIKV African MR766 strain. All of the MV-E2 vaccinated animals survived the challenge. In contrast, the animal with the lowest ZIKV neutralizing titer (Supplementary Fig. 3d) in the MV-E0 group showed signs of ZIKV disease and was euthanized, confirming a certain threshold of neutralizing titer determines protection (Supplementary Fig. 3e). All of the mice in the Prevac groups showed signs of ZIKV disease on day 9, while the controls showed signs of ZIKV disease on day 7 and were euthanized. The Prevac and control groups showed significantly higher ZIKV RNA in the blood, brain, and reproductive tract than the MV-E0 and MV-E2 vaccinated animals (Supplementary Fig. 3f–h). The findings of this study confirm that the MV-E2 vaccine is efficacious irrespective of the route of immunization (Fig. 2, Supplementary Figs. 1–3) and that prior MV immunity affects the efficacy of the MV-E2 and MV-E0 vaccines.

### Rationale, design, and characterization of second-generation MV-ZIKV vaccines

**Fig. 3:**
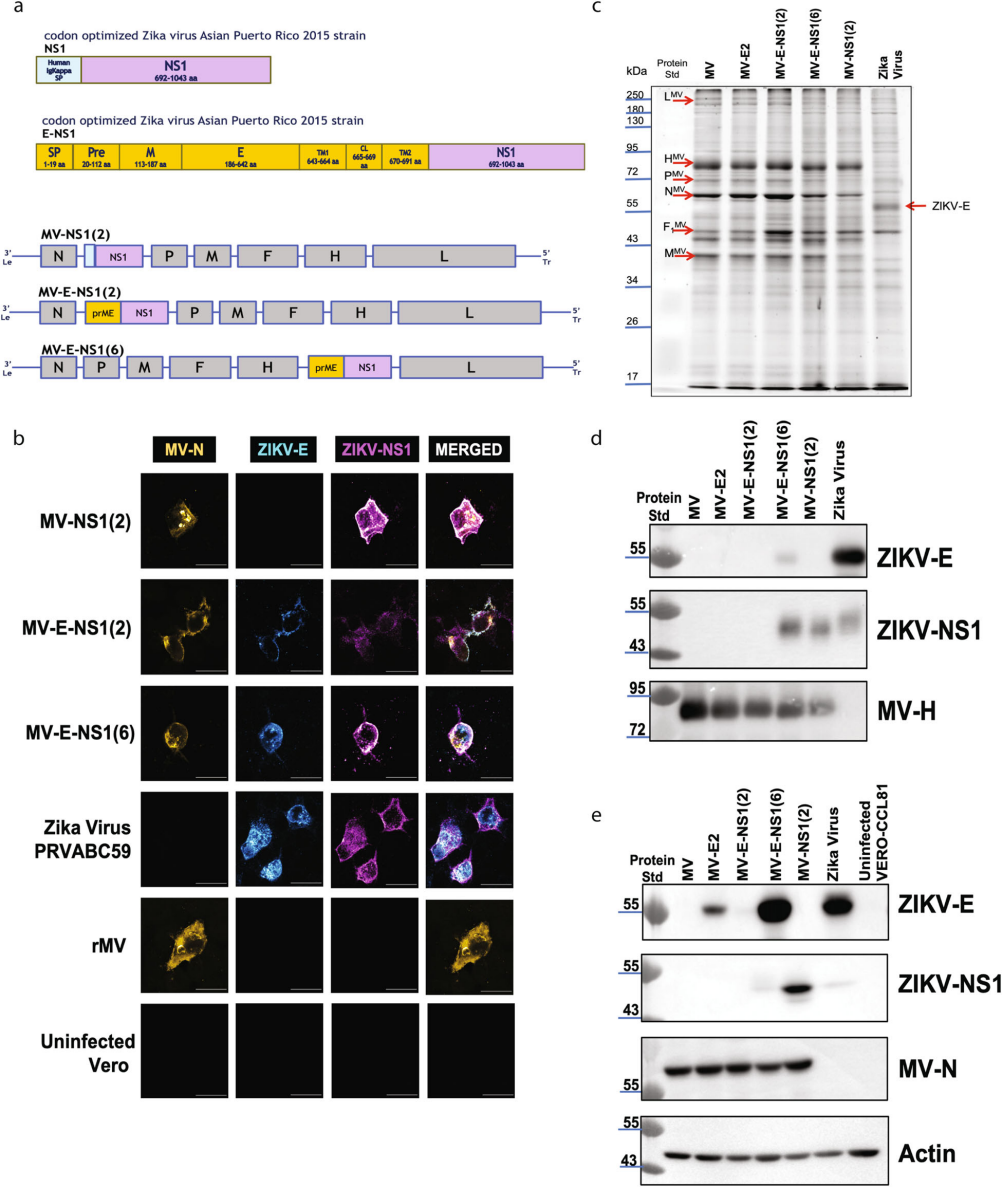
Generation and characterization of second-generation MV-based candidate ZIKV vaccines. **a** Second-generation vaccine constructs. **b** Immunofluorescence staining of Vero cells were infected at MOI-0.1 for 72 h with the recovered modified MV-ZIKV candidate vaccines and control viruses. The permeabilized cells were stained for MV using an α-MV N protein (Sigma, NP-Cl25) mouse monoclonal conjugated with dylight488 (green color changed to Yellow for visualization), for the ZIKV-E using a mouse monoclonal antibody (Biofront-1176-56) and for ZIKV NS1 using human monoclonal antibody EB9. ZIKV-E is stained with a secondary α-mouse AF568 (red color changed to Cyan for visualization) antibody and ZIKV-NS1 is stained with a secondary goat α-human AF-647 (far-red color changed to Magenta for visualization) antibody. Confocal images were taken using NIKON-A1R, 60X magnification, 2.5X zoom. The scale bar measures 30 μm. The data is representing one replicate of *n* = 3. **c** Sucrose-purified virions were analyzed on SDS-PAGE (10%) stained with SYPRO Ruby. Zika virus PRVABC59 was loaded with the control virus. The data is representing one replicate of *n* = 3. **d** Western blot analysis of sucrose purified virions probed for ZIKV-E (Biofront mouse mAb-1176) and MV-H (Rabbit polyclonal sera). Zika virus PRVABC59 was loaded as the control virus. The data is representing one replicate of *n* = 3. **e** Western blot analysis of cell lysates of Vero cells infected with MV-ZIKV candidate vaccine at MOI-5. Zika virus PRVABC59 infected cell lysates were loaded as the control. The blot was probed for ZIKV-E (Biofront mouse mAb), ZIKV-NS1 (Abcam, B4 mouse mAb) and MV-N (Sigma, NP-Cl25). The data is representing one replicate of *n* = 2.

While the MV-E2 vaccine was efficacious when administered, i.p. or i.m., it did not protect the brain and the reproductive tract of vaccinated animals when challenged with the mouse-adapted ZIKV African MR766 strain. Other researchers have shown that antibodies directed towards NS1 expressed on the surface of infected cells can protect via antibody-dependent cellular cytotoxicity (ADCC) or antibody-dependent cellular phagocytosis (ADCP)^13^. We, therefore, generated MV-NS1(2) that expressed ZIKV NS1 from ATS-2 and exchanged the ZIKV signal peptide (SP) with that of the human Ig Kappa signal peptide to allow for better secretion^13^. Additionally, we generated constructs that expressed the prME-NS1 from ATS-2 and ATS-6, naming them MV-E-NS1(2) and MV-E-NS1(6), respectively, to test whether the NS1 antibodies enhanced the protection elicited by ZIKV-E antibodies (Fig. 3a). We recovered these second-generation MV-ZIKV vaccines using the standard methods described in the Methods section.

The recovered second*-*generation MV-ZIKV viruses were assessed for their expression of ZIKV-E, ZIKV-NS1, and MV-N by immunofluorescence assay (Fig. 3b). The MV-NS1(2) co-express MV-N and ZIKV-NS1, while MV-E-NS1(2) and MV-E-NS1(6) express MV-N, ZIKV-E, and ZIKV-NS1 proteins. The control ZIKV PRVABC59-infected cells were stained for ZIKV-E and ZIKV-NS1, while the empty vector rMV (Edmonston B strain) was only stained for MV-N.

To investigate the influence of the foreign gene on virus production, we assessed multi-step virus growth kinetics of MV-ZIKV vaccines (Supplementary Fig. 4). Released and cell-associated viruses were harvested at all time points. The MV-E2 and the MV-E-NS1(6) viruses reached similar peak titers but were lower than the empty MV vector, while the MV-NS1 yielded slightly higher titers to the MV-E2 virus on day 4. In contrast, the MV-E0 yielded significantly lower titers than MV. For the single antigen (ZIKV-E or NS1), viruses’ peak titers were seen at 96 or 120 hpi, while for the dual antigen vaccines, peak titers were seen at 120 hpi. The MV-E-NS1(6) grew to significantly higher titers than MV-E-NS1(2).

Next, the rMV-ZIKV viruses were purified over a 20% sucrose-cushion and analyzed by SDS-PAGE (10%). The Sypro-ruby stained gel showed the presence of all six MV structural proteins in the MV-NS1(2), MV-E-NS1(2), and MV-E-NS1(6) viruses, similar to empty vector rMV and MV-E2 viruses (Fig. 3c). Western blot analysis of the similar SDS-PAGE of purified rMV-ZIKV vaccines probed for ZIKV-E showed an absence of ZIKV-E in all constructs except MV-E-NS1(6) (Fig. 3d) in the virion. ZIKV-E and -NS1 may sometimes co-purify with the virions due to the purification method, as seen by the faint ZIKV-E band in MV-E-NS1(6), as well as NS1 bands seen in the MV-E-NS1(6), MV-NS1(2), and Zika virus virions (Fig. 3d). The co-purification of ZIKV-E was sometimes observed for MV-E2 and MV-E0 viruses. The western blot of sucrose purified virions showed the presence of MV-H in all MV’s except for the control Zika virus virions (Fig. 3d).

Cell lysates obtained from Vero cells infected by rMV-ZIKV vaccines at an MOI of 5 for 60 h were probed for ZIKV-E, ZIKV-NS1, and MV-N in a western blot (Fig. 3e). The western blot confirmed the expression of ZIKV-E in MV-E-NS1(6) and MV-E2, with greater expression seen for the MV-E-NS1(6) virus. A faint ZIKV-E band is seen in the MV-E-NS1(2) cell lysates, which correlates with its slow replication (Fig. 3e, Supplementary Fig. 4). We observed the highest ZIKV-E expression in the MV-E-NS1(6) construct that the MV-E2 or the MV-E-NS1(2) when infecting Vero cells. While this data contradicts the MV transcription gradient, we observe higher in-vitro expression consistently for the ZIKV gene(s) inserted more distal from the transcription start at the 3′ end for unknown reasons. In addition, replication kinetics is affected by the size of the insert and the insertion point in the absence of interferon (Vero). A similar result was seen for eGFP expressed on Vero cells compared to interferon-producing MRC-5 cells^30^. The ZIKV-NS1 expression is the highest in the MV-NS1(2) cell lysates, with low levels seen in the MV-E-NS1(6) and the control Zika virus-infected cell lysates. The low levels of ZIKV-NS1in MV-E-NS1(6) seen may be due to efficient secretion of the NS1 out of the cells. No ZIKV-NS1 was seen in the MV-E-NS1(2) cell lysates. Similar MV-N expression was seen in all the recombinant MV cell lysates (Fig. 3e). Similar levels of the actin control were detected in the western blot of the cell lysates (Fig. 3e).

We next wanted to characterize the ZIKV-E and ZIKV-NS1 protein secreted by our MV-ZIKV vaccines. In a western blot, the purified SVPs resuspended in a non-reducing buffer were probed for ZIKV-E and ZIKV-NS1 (Supplementary Fig. 5a, b). The ZIKV-E probed blot showed the presence of a monomeric and a dimeric band similar to the Zika virus made SVP in the MV-E2 and the MV-E-NS1(6) lane, while no band is seen in the controls— empty MV, MV-NS1(2) lanes. In addition, a faint-strong band above 250 kDa was also seen in MV-E2, MV-E-NS1(6), and Zika virus lanes. This suggests that the 1st and 2nd generation MV-ZIKV vaccine generate SVPs similar to the Zika virus. The blot probed for NS1 yielded a smear ranging from 55 kDa to ~70 kDa above the size of the monomeric NS1 (50 kDa), indicating that the NS1 made by the MV-ZIKV vaccines is a combination of monomeric and dimeric (membrane-bound) forms. SVPs for the MV-E0 virus could not be purified and were not included in this blot.

### Efficacy of second-generation MV-ZIKV vaccines using lethal mouse-adapted ZIKV African MR766 strain

**Fig. 4:**
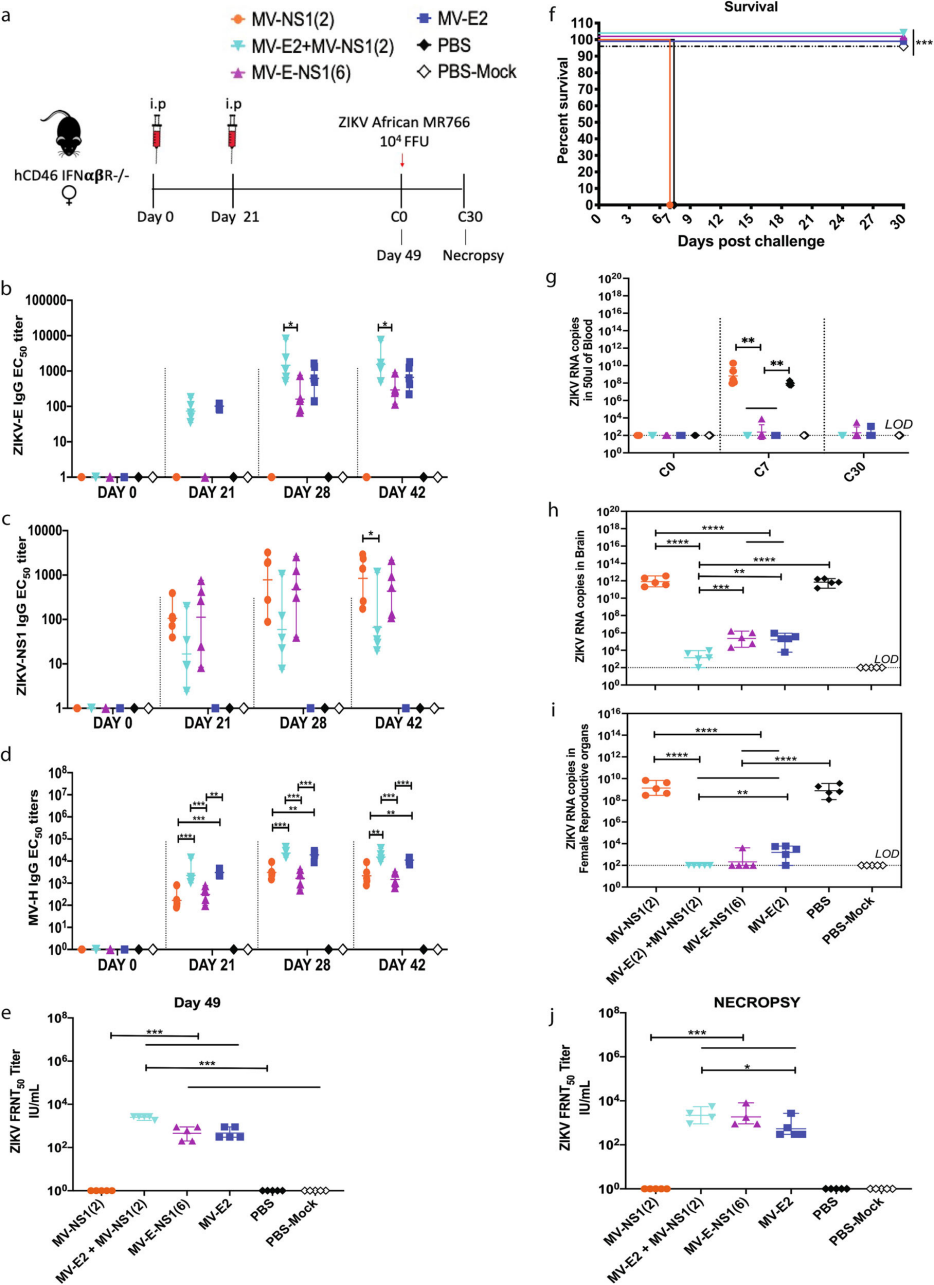
Immunogenicity and efficacy testing of second-generation candidate MV-ZIKV vaccines using a lethal ZIKV African MR766 challenge strain. **a** Timeline of vaccination, challenge, and viral load determinations. Anti-ZIKV-E (**b**), anti- ZIKV-NS1 (**c**), and anti-MV-H (**d**) ELISA IgG EC_50_ titers of the vaccinated animals are plotted on a graph for all animals at different time points. The mean of triplicate EC_50_ values is depicted per animal. Mean ± SD is depicted per group. ZIKV neutralization with PRVABC59 Asian strain. FRNT was performed on day 49 (**e**) and necropsy (**j**) sera from vaccinated animals and controls. The mean 50% neutralizing titer of triplicate values is depicted per animal as IU/mL as ZIKV standards (Bei) were used. The Mean ± SD is depicted per group. **f** Kaplan–Meier survival curve analysis of vaccinated and control animals post-challenge. ZIKV RNA copies by qPCR in the blood (**g**), brain (**h**), and reproductive tract (**i**). The mean of triplicate RNA copies is depicted per animal. The Mean ± SD is depicted per group. The LOD is 100 copies. Statistics for Fig. 4b–e, (**g**–**j**) were done using the one-way ANOVA with post hoc Tukey HSD test and performed on log-transformed data for each time point. Figure 4f, survival curves were analyzed using the log-rank test with a Bonferroni correction. Only significant differences are depicted. *P* value of 0.1234(ns), 0.0332(*), 0.0021(**), 0.0002(***), <0.0001(****) are depicted accordingly. LOD stands for limit of detection. A horizontal line (—) is used to include all groups below it.

Six groups of five female *hCD46 IFNαβR*^*−/−*^ mice (10- to 12-weeks old) each were immunized with 10^5^ TCID_50_ i.p. on day 0 and boosted on day 21 with either MV-NS1(2), combination vaccine group—MV-E2 & MV-NS1(2), MV-E-NS1(6), MV-E2, and two PBS groups (Fig. 4a). The combination vaccine group received 10^5^ TCID_50_ each of MV-E2 & MV-NS1(2) vaccine (2 × 10^5^ TCID_50_ total) (Fig. 4a). The MV-E-NS1(2) was not included in this study because a pilot study with it failed to achieve seroconversion in mice, maybe due to its slow replication (Supplementary Fig. 4). For all time points, the combination vaccine group elicited similar ZIKV-E IgG antibody titers to the MV-E2 vaccine group, with only modestly higher ZIKV-E responses than the MV-E-NS1(6) vaccinated animals (*p* = 0.05) (Fig. 4b). The MV-NS1(2) and the MV-E-NS1(6) elicited similar ZIKV-NS1 antibody titers, while the combination vaccine group had lower ZIKV-NS1 responses (Fig. 4c). The combination vaccine group had similar MV-H responses to the MV-E2 group but significantly higher titers than the MV-NS1(2) and the MV-E-NS1(6) groups (Fig. 4d).

Based on the collected immunogenicity data, we decided to challenge the mice on day 49 with 10^4^ FFU of ZIKV African MR766 strain s.c., except for one PBS group that was mock challenged with PBS. As expected, the combination vaccine group MV-E2 & MV-NS1, the MV-E-NS1(6) group, and the MV-E2 group survived the challenge, showing no signs of ZIKV disease (Fig. 4f). No detectable ZIKV RNA was seen in the blood of the combination vaccine group, while one animal in the MV-E2 and the MV-E-NS1(6) groups showed the presence of ZIKV (Fig. 4g). Significantly lower ZIKV RNA was seen in the brains and female reproductive organs (Fig. 4h, i) of the MV-E2 and MV-E-NS1(6) mice than in the MV-NS1(2) mice and the PBS mice that were euthanized when they showed signs of ZIKV disease. The combination group had significantly lower viral RNA in the brains and complete viral clearance in the female reproductive organs than the MV-E2 vaccinated animals. The MV-E-NS1(6) vaccinated animals achieved almost complete viral clearance in the female reproductive tract except for one animal. The viral clearance achieved by the combination vaccine MV-E2 & MV-NS1 can be correlated with the highest ZIKV neutralizing antibodies (Fig. 4e & j). Lower ZIKV neutralizing antibodies were observed in the MV-E2 and the MV-E-NS1(6) than the combination vaccine group, and as expected, no neutralizing activity was seen in the MV-NS1(2) group. The presence of NS1 antibodies in the combination vaccine and MV-E-NS1(6) vaccine group provided better viral clearance in the female reproductive tract than MV-E2 alone (Fig. 4i). One animal from the combination vaccine and the MV-E-NS1(6) group died due to unrelated reasons on day 30.

### The Measles Zika vaccines induce durable responses

**Fig. 5:**
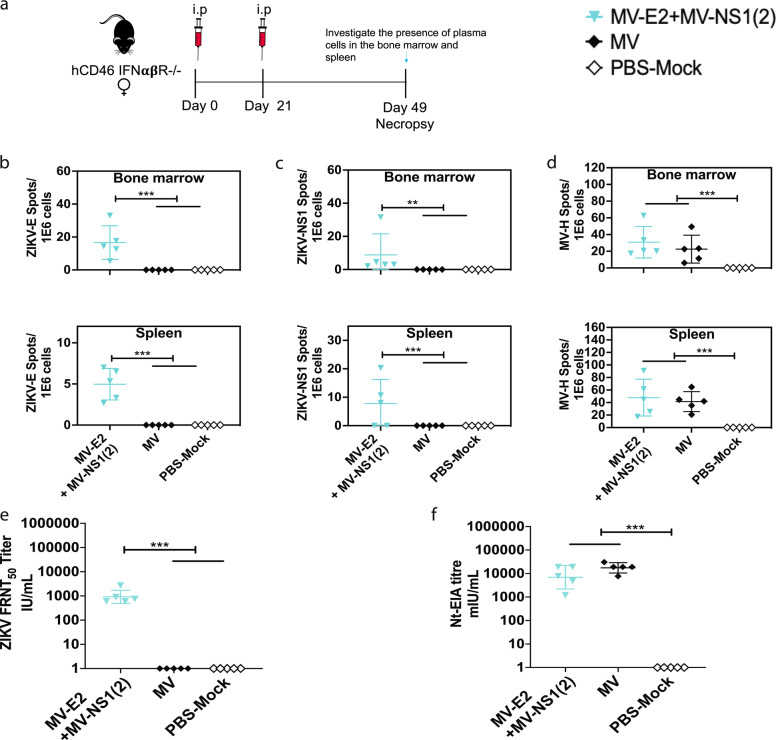
Plasma cell responses induced by the MV-ZIKV vaccines. **a** Timeline of vaccination, bone-marrow and spleen harvesting. ZIKV-E specific (**b**), ZIKV-NS1 specific (**c**), and MV-H specific (**d**) Plasma cells in the bone marrow and spleen. The mean of triplicates is depicted for each animal. Mean ± SD is depicted per group. **e** ZIKV Neutralization with PRVABC59 Asian strain. FRNT was performed on necropsy sera from vaccinated animals and controls. The mean 50% neutralizing titer of triplicate values is depicted per animal as IU/mL as ZIKV standards (Bei) were used. The Mean ± SD is depicted per group. **f** MV Neutralization with low-passage MV Edmonston strain. Nt-EIA assay was performed on necropsy sera from vaccinated animals and controls. The mean of 50% neutralizing titer (Nt-EIA titers) of triplicates is plotted per animal on the graph in terms of mIU/mL as bei standards were used. The Mean ± SD is depicted per group. Statistics for Fig. 5b–f were done using the one-way ANOVA with post hoc Tukey HSD test and performed on log-transformed data for each time point. *P* value of 0.1234(ns), 0.0332(*), 0.0021(**), 0.0002(***), <0.0001(****) are depicted accordingly. A horizontal line (—) is used to include all groups below it.

We tested the potential of two of our rMV-ZIKV vaccines, MV-E2, and MV-NS1, to induce long-term immunogenicity by investigating the presence of plasma cell (PC) responses. Three groups of five female *hCD46 IFNαβR*^−/−^ mice (10- to 12-weeks old) were immunized i.p. with the 2 × 10^5^ TCID_50_ of the combination vaccine, MV-E2 & MV-NS1(2) (i.e., 10^5^ TCID_50_ of each virus), rMV, or PBS on day 0 and boosted on day 21. Their bone marrow and spleen were harvested on day 49 and assessed for the presence of ZIKV-E-, ZIKV-NS1-, and MV-H-specific PC’s. (Fig. 5a). High ZIKV-E and MV-H IgG responses were seen in the combination vaccine group, while ZIKV-NS1 IgG responses were variable (Supplementary Fig. 6a–c). ZIKV-E specific PCs were detected in the bone marrow and the spleen of the combination vaccine group (Fig. 5b). Similar to the low-level ZIKV-NS1 antibody responses (Supplementary Fig. 6c), the ZIKV-NS1 PCs were also low-level (Fig. 5c). High ZIKV neutralizing titers were induced in the combination vaccine group (Fig.5e). Additionally, the MV-H specific PCs in the combination group were similar to those of the empty vector rMV group, indicating that the addition of ZIKV-E and NS1 did not affect the vector response (Fig. 5d). These results suggest that the MV-ZIKV vaccines can induce durable responses.

### Measles virus neutralizing titers induced by the Measles Zika vaccines

Sera from the previous experiment (Fig. 5) were assessed for the presence of MV-neutralizing titers. To test the potential of the vector, we chose the combination vaccine for this analysis that included two of our MV-ZIKV vaccines. The combination group induced similar measles neutralizing titers to the empty vector rMV group (Fig. 5f), indicating that measles immunity was unaffected by the addition of ZIKV-E and NS1.

### NS1 antibodies play a role in fetal protection in pregnant mice when challenged with a lethal mouse-adapted Zika African MR766 strain

**Fig. 6:**
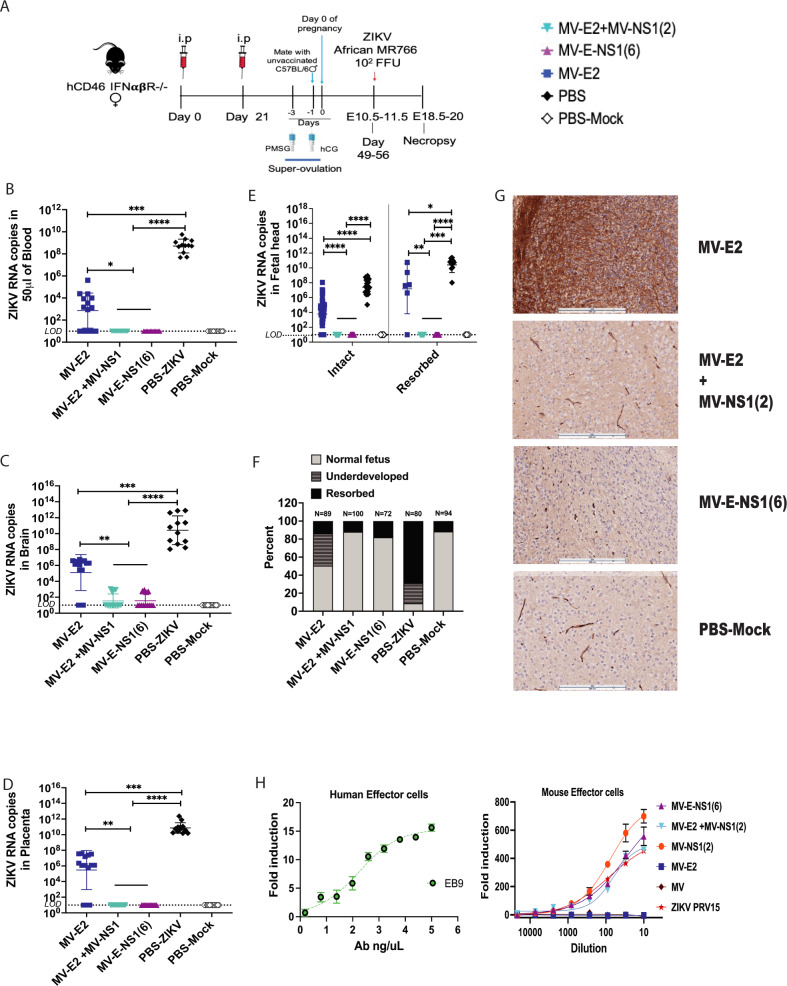
NS1 antibodies play a role in fetal protection in pregnant mice when challenged with a lethal mouse-adapted Zika African MR766 strain. **a** Timeline of vaccination, super-ovulation and challenge. Number of pregnant animals per group: MV-E2 = 14; MV-E2 + MV-NS1 = 14; MV-E-NS1(6)=13; PBS = 12 and PBS-Mock-12. ZIKV RNA copies by qPCR in: the blood (**b**), brain (**c**) and placenta (**d**) of the pregnant mice, (**e**) in the intact fetal heads and resorbed fetuses. The mean of triplicate RNA copies is depicted per animal. The Mean ± SD is depicted per group. The LOD is 10 copies. **f** Percentage of resorbed, underdeveloped and normal fetuses per group. No of pups for each group: MV-E2 group: Normal fetus (Nf) = 45, underdeveloped (u) = 32, resorbed (res); MV-E2 + MV-NS1: Nf=88, res=12; MV-E-NS1(6): Nf=59, res=13; PBS-ZIKV: Nf=7, u = 18, res=55; PBS-Mock: Nf=84 res=11. **g** Fetal brains stained for Zika virus NS1 antibodies using human monoclonal EB9. The figure represents one replicate of five-six fetal heads for each group except for PBS-ZIKV. PBS-ZIKV fetal heads could not be stained as the tissue was friable. The scale bar = 200 μM. **h** ADCC activation activity luciferase reporter assay. ZIKV PRVABC59 infected Vero cells were incubated with sera or control antibody EB9 (Human anti-Zika-NS1 ab), followed by incubated with effector cells (mouse FcγRIV or human FcγRIIIa expressing Jurkat cells) at ratio of 5:1. Heat inactivated pooled sera was used for the assay. The figure represents one replicate of 4 repeats. Fold induction is depicted for each group. Statistics for Fig. 6b–e were done using Pairwise Two-Sided Multiple Comparison Analysis of log-transformed data by the Dwass, Steel, Critchlow-Fligner method. Only significant differences are depicted. *P* value of 0.1234(ns), 0.0332(*), 0.0021(**), 0.0002(***), <0.0001(****) are depicted accordingly. LOD stands for limit of detection. A horizontal line (—) is used to include all groups below it.

Based on results indicating that the combination of MV-E2 and MV-NS1(2) and MV-E-NS1(6) can protect the female reproductive tract (Fig. 4), we tested these vaccines in a pregnant mouse model. Two experimental replicates of five groups of ten female *hCD46 IFNαβR*^−/−^ mice (10- to 12-weeks old) were immunized i.p. with the 2 × 10^5^ TCID_50_ of the combination vaccine, MV-E2 & MV-NS1(2) (i.e., 10^5^ TCID_50_ of each virus) or 10^5^ TCID_50_ of MV-E2 or 10^5^ TCID_50_ of MV-E-NS1(6) and two PBS groups on day 0 and boosted on day 21 (Fig. 6a). Superovulation was induced in the mice by injecting 5 IU/mouse of pregnant mare serum gonadotropin (PMSG; i.p.) on day 36 (day-3 to pregnancy), and 5 IU/mouse of human chorionic gonadotropin (hCG; i.p.) on day 38 (day-1 to pregnancy). Unvaccinated C57BL/6 males were placed in the cage, and the next day was considered day 0 of pregnancy. The mice were checked for plugs, weighed daily, and challenged with 10^2^ FFU of lethal ZIKV African MR766 strain on day 49–56, depending on pregnancy status being embryonic age 10.5–11.5 days humanely euthanized on day 18.5–20. One PBS group was mock challenged with PBS. Combining the two independent experiments, the MV-E2 and the combination vaccine group had fourteen pregnant mice each, the MV-E-NS1(6) had thirteen pregnant mice, the PBS and PBS-mock challenged group had twelve pregnant mice. The ZIKV-challenged PBS group showed signs of severe ZIKV disease at the necropsy time point, while the MV-E2, MV-E-NS1(6), and the combination vaccinated animals showed no signs of disease. The pregnant females in the combination vaccine group and the MV-E-NS1(6) had significantly lower or no viral RNA in the blood, brain, and placenta than the MV-E2 and PBS group (Fig. 6b–d). The MV-E2 group had significantly lower viral RNA in the blood, brain, and placenta than the PBS group (Fig. 6b–d). Fetuses from the combination vaccine and the MV-E-NS1(6) group showed the absence of detectable ZIKV RNA, with 88% (combination) or 81.9% (MV-E-NS1(6)) normal fetuses and the remaining resorbed (Fig. 6e, f). Significantly high ZIKV viral RNA was detected in the intact and resorbed fetuses of the MV-E2 group, with 50.6% normal fetuses, 36% underdeveloped, and 13.4% resorbed (Fig. 6e, f). The fetuses in the PBS group had the highest viral RNA and were mostly resorbed (68.8%) or underdeveloped (22.5%) with a few normal-sized pups (8.7%). ZIKV viral RNA was undetectable in the fetuses of the mock challenged PBS group, with 88.4% normal fetuses and 11.6% resorbed. The resorbed fetuses had higher viral RNA copies than intact fetuses for the PBS and MV-E2 groups (Fig. 6e).

Lastly, five-six fetal heads were randomly taken from each group to be stained for ZIKV NS1 using a human monoclonal antibody EB9. The absence of viral RNA in the combination and MV-E-NS1 (6) group was confirmed with the lack of NS1 antigen staining in both groups (Fig. 6g). The MV-E2 showed a varying degree of ZIKV NS1 antigen staining in the fetal heads (Fig. 6g). We could not stain the fetal heads from the PBS challenged group as they were friable.

### NS1 antibodies elicit antibody dependent cellular toxicity functions

Despite the induction of high ZIKV neutralizing antibodies in MV-E2, MV-E-NS1(6) groups, and the combination vaccine (Fig. 4e), NS1 antigen-based vaccines demonstrated improved fetal protection in the presence of ZIKV-E (Figs. 4i and 6e–f). To understand the mechanism of NS1 antibody mediated protection, we tested mouse sera and controls for antibody mediated cellular cytotoxicity (ADCC) activity using ZIKV PRVABC59 infected Vero cells in an ADCC activation Reporter Bioassay where the Fc portion of the antibody binds to the FcγRIIIa (mouse) or FcγRIV (human) of the effector cells (Jurkat) results in a quantifiable luminescence signal from NFAT (nuclear factor of activated T-cells) pathway. We observed the highest ADCC activity in the MV-NS1(2) serum (Fig. 6h), which also had the highest NS1 antibody response (Fig. 4c). The serum from the combination vaccine, MV-E-NS1(6) vaccine, and ZIKV PRVABC59 infected mice elicited similar ADCC activity. MV-E2 and MV sera induced no detectable ADCC activity. The available control antibody human anti-ZIKV-NS1 EB9 induced high ADCC activity as previously observed^31^.

## Discussion

Several candidate ZIKV vaccines are in Phase 1 clinical trials, with the most advanced being the DNA, mRNA, live-attenuated Zika virus (rZIKV/D4Δ30-713), and the alum adjuvanted Zika purified inactivated virus vaccines^14^. Both the DNA vaccine and the inactivated virus vaccines are safe platforms that have been well-tolerated in humans^15,17^. Despite these results, recent studies with the same DNA vaccine found that it did not completely prevent adverse fetal outcomes in pregnant monkeys under prolonged ZIKV exposure^22^. Failure of the Zika purified inactivated virus (ZPIV) to induce durable responses in human phase I clinical trials beyond 16 weeks suggested that further development in ZIKV vaccine strategies is paramount for protecting susceptible pregnant women and their unborn fetuses^32^. We tested the effectiveness of our dual antigen vaccines, the combination vaccine, and the MV-E-NS1(6) vaccine in a pregnant mouse model, and our findings indicated that NS1 antibodies are required to protect the female reproductive tract and their unborn fetuses.

There have been concerns that a ZIKV vaccine induces cross-reactive E protein-specific (primarily envelope domain I/II, fusion loop epitope-FLE) antibodies shared across flaviviruses and can potentiate antibody-dependent enhancement (ADE). ADE has been described among the dengue virus (DENV) strains and has had implications for developing a DENV vaccine. Several in-vitro and in-vivo mouse studies have confirmed ADE of ZIKV with DENV antibodies and vice versa^33^. ADE caused by the presence of DENV antibodies has been speculated to be one of the reasons for ZIKV-induced microcephaly^34^. The hypothesis that transcytosis of IgG-virion complexes can occur across the placenta by utilizing the neonatal Fc receptor (FcRn) emerged from in-vitro studies in cytomegalovirus (CMV) and HIV^35^. In-vitro studies of ZIKV enhancement in human placental tissue in the presence of DENV antibodies strengthened the hypothesis^36^. Human and monkey studies have indicated that prior DENV exposure provides some cross-protection to ZIKV infection and vice versa^37–39^. However, a recent study showed that prior Zika virus infections might enhance the future risk of severe dengue disease^40^.

Different ZIKV strains have varied pathogenicity, the Asian PRVABC59 strain is non-pathogenic, and the African MR766 strain is highly lethal in *IFNαβR*^−/−^ mice. The African MR766 strain is also the most potent at causing brain damage and postnatal lethality in mice^41^. We investigated the immunogenicity and efficacy of MV vaccine vector-based ZIKV vaccines in *hCD46 IFNαβR*^−/−^ mice. The first-generation ZIKV vaccines, both expressing the full-length preME, MV-E0, and the MV-E2 vaccines, induced high ZIKV-E neutralizing antibodies and were protective when challenged with the non-lethal Asian PRVABC59 (10^6^ FFU) strain and the lethal African MR66 (10^4^ FFU) strain. Both vaccines achieved neutralizing titers but did not achieve complete viral clearance in the lethal challenge model^42^. The ZIKV-3′UTR-LAV-, GAd-Zvp-, and the E-dimer-vaccinated mice had similarly incomplete ZIKV clearance in organs when challenged with another ZIKV African strain (Dakar)^21,43–45^.

Recent studies have indicated that ZIKV NS1 antibodies and T cell responses play protective roles^31^. We found that the NS1 antibodies themselves did not protect the mice from the lethal African MR766 challenge, as the MV-NS1(2) vaccinated animals succumbed to ZIKV disease. Similar results were observed in NS1-DNA vaccinated animals^46^. Both the MV-E2 and MV-E-NS1(6) mice survived the challenge and showed significantly lower ZIKV RNA in the blood and brain of these mice compared to the PBS mice. We observed almost complete viral clearance in the female reproductive tract MV-E-NS1(6) vaccinated mice. At the same time, the combination group completely cleared ZIKV RNA from the reproductive tract, suggesting that NS1 antibodies played a role in this enhanced protection. The combination and MV-E-NS1(6) vaccines also protected the fetuses in a lethal pregnant mouse model. In addition, the induction of ZIKV-E and ZIKV-NS1 PC responses were seen in the combination vaccine group. Such durable responses have only been observed in the GAd-Zvp vaccinated animals that showed the presence of ZIKV-E LLPC’s and memory B cells^43^. Taken together, the combination vaccine, MV-E2 & MV-NS1(2) and MV-E-NS1(6) are the improved measles vaccines to provide complete fetal protection and viral clearance in the placenta when challenged with the African MR766 strain.

Past studies had shown that high E-specific neutralizing titers were predictive of protection for other flaviviruses. However high ZIKV-E specific neutralizing was not sufficient to completely prevent adverse fetal outcomes in mice and monkeys for several candidate vaccines^21,23,44^. Comparative studies of soluble preME (ΔStem/TM) and full-length preME as the immunogen using either the DNA vaccine or gorilla adenovirus vector (GAd) or VSV vector indicated that the full-length prME might provide better protection in terms of controlling ZIKV replication^43,47–49^. The full-length preME assembles into a subviral particle (SVP) while the preME (ΔStem/TM) is secreted as a soluble E protein alone which may affect the epitopes exposed and thereby affect the quality of antibodies generated^50^. The preliminary characterization of SVP of the MV-ZIKV vaccines that express either prME or prME-NS1 suggests that it may make ZIKV-E monomers and dimers like the Zika virus. Further analysis would be required to verify the structure of the SVP expressed by the MV-ZIKV vaccines.

Our findings add to the knowledge base on how a vaccine may be designed to provide complete fetal protection against ZIKV. Previously tested mRNA, DNA, GAd-Zvp, and Ad26 vaccines using the preME or preME-FLE (ZIKV-E fusion loop epitope mutant) as the antigen have been found to induce very high ZIKV neutralizing titers but could not achieve complete placental and/or fetal protection in the vaccinated pregnant mice and primates^21–23,43,51^. The Ad26 ZIKV vaccine has shown the most promising results in human clinical trials, inducing durable neutralizing antibodies and ZIKV-E specific cellular responses. The limitation is that the study was conducted in a non-endemic flavivirus area^52^. An exception to this finding was demonstrated in the Jagger et al. study, which observed complete placental and fetal protection in an mRNA-LNP (encoding prME) vaccinated hSTA2-KI mouse model using the MA-ZIKV Dakar 41525 strain. This may be due to the adjuvating potential of the LNP, reduced viremia induced by the MA-Dakar 41525 strain, and the immunocompetent model allowing for adequate innate immune responses to control viral spread^53^. ZIKV-E FLE antibodies have demonstrated ADE activity in-vitro and in mice and are suspected to be causing adverse fetal outcomes. An alternative approach of using two DIII (domain III is the receptor-binding domain) monoclonals was investigated and found to reduce fetal pathology in primates but it did not prevent maternal viral spread^54^. Another study showed that a measles-based vaccine expressing preME (ΔStem/TM) -MV-ZIKA did not clear ZIKV from the placenta of the vaccinated animals in a low-dose Asian strain challenge in an hCD46-IFNAR^−/−^ model but cleared the virus from the fetuses, verifying the importance of full-length preME as the immunogen^55^. Following the conclusion of the clinical trial of the MV-ZIKA vaccine (NCT02996890) in 2018 by Themis Bioscience, they modified their construct to include the stem and TM (MV-ZIKA-RSP, European Patent Office - EP3184118A1). MV-ZIKA-RSP is similar to our MV-E2 construct in the antigen but differs in the insert position. While no animal studies have been published with MV-ZIKA-RSP, the vaccine had completed phase1 clinical trials (NCT04033068) in June 2020, but its results are not yet publicly available.

NS1 antibodies and T cell responses provided partial protection or protection in low-dose challenge models using the VSV and DNA vaccine platforms, but the MVA-NS1 vaccine was the only NS1 vaccine to protect against a lethal African strain challenge in CD-1/ICR mice^13,20,46,48^. The two LAVs, 3′UTR-∆10-LAV and the ZIKV-NS1-DKO (4 amino acid substitutions in the NS1 glycosylation sites), were tested in pregnant mouse models, and the 3′UTR-∆10-LAV protected pregnant mice from the Asian PRVABC59 strain while the ZIKV-NS1-DKO failed to clear the ZIKV from fetal brains. Neither LAV study reported the NS1-specific antibodies or T cell responses induced by the vaccine^21,56^. In addition, the DNA vaccine that protected non-pregnant monkeys in an Asian PRVABC59 strain challenge failed to completely prevent maternal viremia and adverse fetal outcomes in pregnant monkeys^22,57^. The Wessel et al. study described the role of NS1 antibodies in pregnancy wherein several mouse and human NS1 monoclonal antibodies rendered significantly reduced viral loads in the placenta and fetal heads compared to isotype controls when challenged with a ZIKV African (mouse-adapted Dakar) strain^58^. Human studies on maternal antibodies of microcephalic newborns have observed an increase in ZIKV neutralizing capacity, with antibodies directed towards EDIII and lateral ridge EDIII antibodies compared to control newborns without microcephaly. This study also highlighted those mothers of microcephalic infants developed much lower NS1 antibodies than control newborns without microcephaly^59^. Conversely, another study by the same investigators showed that antibody responses in individuals who developed high anti-ZIKV neutralizing antibodies had high EDIII and EDIII lateral ridge epitope antibodies^60^. The two Robbiani et al. results congruently verify our hypothesis that both ZIKV-E and NS1 responses are needed for placental and fetal protection. To further corroborate this theory, high ZIKV NS1 antibody responses were observed in healthy Thai people who had high ZIKV neutralizing antibodies^12^.

Collectively, our study and the published data suggest that ZIKV-E neutralizing antibodies protect non-pregnant mice and monkeys, while NS1 antibodies cannot provide such protection by themselves. Two recent publications, utilizing the VSV-NS1 vaccine as well as testing a broadly protective flavivirus NS1 monoclonal antibody, demonstrated partial protection of NS1 antibodies in a lethal challenge model^61,62^. However, the MVA-NS1 vaccine tested in CD-1/ICR mice provided 100% protection when intracranially challenged with the ZIKV African MR766 strain. Although comparison across vaccine candidates cannot be made as ZIKV-specific protection differ dramatically across candidates and that the induction of high neutralizing antibody titers may not be a good predictor of protection but rather neutralizing antibodies to mature ZIKV virions may be the most critical^63^. While ZIKV-E neutralizing antibodies are essential, the NS1 antibodies and T cell responses may aid its faster viral clearance^46,64^. More importantly, CD4^+^ T cells were recently identified as playing a vital role in protecting from ZIKV-induced neurologic disease and viral control^65^. While the measles vaccine induces robust CD4^+^ and CD8^+^ T cell responses in infants (>6 months) and adults, the CD4^+^ and CD8^+^ T cell responses are yet to be examined for the MV-ZIKA dual antigen vaccines^66^. The MV-ZIKA vaccine-induced T cell responses may play an essential role in the protection and will be assessed in a follow-up study. Despite having high neutralizing antibodies in the MV-E2 group, better protection was observed in the MV-E0 vaccinated animals suggesting that MV-based vaccines may be inducing cellular responses that may play a role in protection. The low titer or incomplete seroconversion against NS1 induced by the combination vaccine may require further adjustment in the vaccine titers of the MV-E2 and MV-NS1(2) vaccines. The dual antigen vaccine, MV-E-NS1(6) is a robust vaccine as it is stable for production and inducing antibody responses. Production and safety of the combination vaccine and the MV-E-NS1(6) vaccine should follow the precedent of the measles vaccine. However, it may be more difficult for the combination vaccine to pass the rigorous manufacturing and approval process to reach the market. This study provides substantial evidence demonstrating the role of dual antigens, ZIKV E and NS1, in providing female reproductive tract protection and is thereby a promising ZIKV vaccine candidate that warrants further preclinical development.

## Methods

### Experimental design

Five *hCD46 IFNαβR*^−/−^ (IFNARCD46tg) breeding pairs were received from Dr. André Lieber (University of Washington, Seattle). Both male and female *hCD46 IFNαβR*^−/−^ mice were used in this study. For the pregnant mouse model, *hCD46 IFNαβR*^−/−^ mice were housed individually in microisolator cages. In total, 8–10 weeks-old C57BL/6 male mice were purchased from Charles River for the pregnant mouse model.

Immunizations were conducted by inoculating mice with vaccines in 100 μl via intraperitoneal (i.p) or intramuscular route (i.m., 50 μl into each gastrocnemius muscle). ZIKV challenges were performed by subcutaneous inoculation in the hind limb with 10^4^ FFU of the mouse-adapted ZIKV African MR766 strain or 10^6^ FFU of the ZIKV Asian (PRVABC59 strain) in 100 μl PBS in non-pregnant mice. For the pregnancy experiments, super-ovulation was induced as the *hCD46 IFNαβR*^−/−^ female mice produce 5–6 pups under normal breeding conditions. *hCD46 IFNαβR*^−/−^ female mice were i.p. injected with 5IU/mouse of pregnant mare serum gonadotropin (PMSG) on day 36 and 5IU/mouse of human chorionic gonadotropin (hCG) on day 38 to induce super-ovulation. Super-ovulated *hCD46 IFNαβR*^−/−^ females were mated with naive wild-type C57BL/6 male mice on day 38. Females were checked for plugs the next day (day 39) and weighed daily until the end of the experiment. At E10.5-11.5, C57BL/6 male mice were removed from the cage, and pregnant dams (*hCD46 IFNαβR*^−/−^) were inoculated with 10^2^ FFU of the mouse-adapted ZIKV African MR766 strain by subcutaneous injection in the hind limb. Animals were sacrificed at E18.5-20, and placentas, fetuses, and maternal tissues were harvested. All African challenged mice were euthanized when ethically defined clinical endpoints were reached (hind-limb paralysis). Mice were randomly allocated to groups. All experiments had five mice per group, except for the pregnant mouse study with twenty mice per group.

### Animals and care

This study was carried out in strict adherence to recommendations described in the Guide for the Care and Use of Laboratory Animals and guidelines of the National Institutes of Health, the Office of Animal Welfare, and the United States Department of Agriculture. All animal work was approved by the Institutional Animal Care and Use Committee (IACUC) at Thomas Jefferson University (animal protocol 01155 and 01873). All procedures were carried out under isoflurane anesthesia by trained personnel under the supervision of veterinary staff. Mice were housed in cages, in groups of five, under controlled conditions of humidity, temperature, and light (12-h light/12-h dark cycles). Food and water were available ad libitum.

### Cells

Vero-CCL81, Vero-E6, and 293 T/T17 cells were purchased from ATCC and maintained in high glucose Dulbecco’s modified Eagle’s medium (DMEM, Corning, 10-017-CV) supplemented with 5% fetal bovine serum (FBS, R&D SYSTEMS, S11150) and 1% penicillin/streptomycin (P/S, Gibco, 15140122) and cultured at 37 °C with 5% CO_2_.

### Viruses

The following ATCC viral stocks were purchased for this study. Zika virus African MR766 strain (ATCC, VR-1838), Zika virus Asian PRVABC59 strain (ATCC, VR-1843), and Measles virus low-passage Edmonston strain (ATCC, VR-24).

### Antibodies

The following antibodies were used in this study: Anti-ZIKV-E mouse monoclonal antibody (Biofront Technologies, 1176-56), Pan-Flavivirus-E 4G2 mouse monoclonal antibody produced from hybridoma cell line D1-4G2-4-15 (ATCC, HB-112), Anti-Measles Nucleoprotein mouse monoclonal antibody produced from hybridoma NP.cl25 (Millipore Sigma, Cat # 95051114), Anti Measles H polyclonal Rabbit sera from Dr. R.Cattaneo.

### Recombinant MV-ZIKV vaccines plasmid construction

All Measles virus clones (Edmonston B strain) used in this study are derived from a full-length cDNA clone (MVeGFP) provided by Roberto Cattaneo (Lars Hangartner, MSc Thesis, University of Zurich; Paul Duprex et al. JVI 1999)^67^. Modified versions of this vector containing additional transcription sites (ATS) between the N and P genes and between the H and L genes were constructed in a pBluescript vector backbone (Agilent). The newly constructed vectors are flanked by a hammerhead ribozyme designed to cleave at the 5′-terminal nucleotide of the antigenome RNA. A DNA fragment containing the hammerhead ribozyme preceded by a T7 RNA polymerase promoter and flanked by BsmBI and NotI sites at the 3′-end was assembled by PCR using primers 5′-GTGTGCGCGCCGTTAATACGACTCACTATAGGGAGACCCAAGCTGGCTAGCTTTGTGGTCTGATGAGTCCCGTGAGGA-3′ and 5′-ACAAGCGCGCGGCCGCACACGACGTCACACGTCTCAGACCCGGTACGCCGGGTTTCGTCCTCACGGGACTCATCAGACCA-3′ and inserted into the BssHII sites of the pBluescriptII vector (Agilent). To remove the GFP gene inserted upstream of the nucleoprotein gene in the original Measles vector, an overlap extension PCR product containing the leader sequence and the nucleoprotein gene was generated using four primers 5′-GTGTGGTCTCTGGTCACCAAACAAAGTTGGGTAAGGATAGTTCA-3′, 5′-AAGTGTGGCCATCTCGGATATCCCTAATCCTGCTCT-3′, 5′-GGATTAGGGATATCCGAGATGGCCACACTT-3′, 5′-GTGTGCGGCCGCTATAACGCGTATCACTCTGTGTGGACCTGGTTCCTAAGTTTTTTATAACAATG-3′ and joined to the hammerhead ribozyme using a golden gate cloning strategy. The PCR fragment also inserted transcription start and stop sequences and additional MluI and NotI sites. A second set of transcription start stop sequences plus additional AatII and AsiSI restriction sites and the entire P gene extending up to the SalI site in the P-M intergenic region was then amplified using primers 5′-GTGTACGCGTTCCTGACGTCTTGCGATCGCGTGCGAGAGGCCAGAACAACA-3′, 5′-GTGTGCGGCCGCAGGTTGTACTAGGTGGGTC-3′ and inserted into the MluI and NotI sites. In the final step, a cDNA fragment extending from the SalI site to the NotI site downstream of the Hepatitis delta virus ribozyme was cut out from MVeGFP and ligated into the newly assembled subclone containing the N and P genes to construct a full-length clone containing an ATS inserted into the N/P intergenic region. This vector served as the backbone for the position 2 vectors described in this manuscript. In addition, was used to construct a bare Measles vector without any additional transcription units (pCW658) by replacing the N-P intergenic region with the intergenic region of the MVeGFP clone. The bare MV clone was then used to insert an additional transcription stop and start sequences and singular restriction sites into the H/L intergenic region by first subcloning the PacI-SpeI fragment, then inserting a synthetic DNA fragment containing stop and start sequences plus MluI, BsiWI, and AsiSI sites between the XmaI and SpeI sites and finally replacing the PacI-SpeI fragment in CW658 with the modified fragment. The resulting vector served as the backbone for the position 6 (ATS-6) vectors described in this manuscript.

To generate the first-generation MV-ZIKV vaccines the codon-optimized Zika prME gene (signal peptide= MR766 strain, GenBank: MK105975.1 and prME sequence= PRVABC59 Asian 2015; GenBank: KU501215.1) was synthesized by GenScript. PCR amplification of the coding region of Zika prME from the gene synthesized plasmid was performed using primers ZMP Fwd1 (5′- GTGTCGACGCGTGGAATCCTCCCGTACGGCCACCATGGGGGCTGATACAAGCATTGGCA - 3′) and ZMP Rev1 5′- GTGTCGGACGTCATTTATGCGGACACTGCGGTGGACAGAAAA-3′. PCR fragments were digested with the respective enzymes and ligated into the ATS-0 or ATS-2 MV vector to generate MV-E0 and MV-E2, respectively (JM109 *E.coli*).

To generate the 2nd generation MV-ZIKV vaccines, the codon-optimized Zika prME-NS1 gene (strain PRVABC59 Asian 2015; GenBank: KU501215.1) was synthesized by GenScript. PCR amplification of the coding region of Zika prME-NS1 from the gene synthesized plasmid was performed using primers MV-ZprME-NS1(2) Fwd 5′-ACAGAGTGATACGCGTACGGGCCACCATGGGGG-3′ and MV-ZprME-NS1(2) Rev 5′-GCACGCGATCGCAAGACGTCGGCTATGCTGTCACC-3′. PCR fragment was inserted into the ATS-2 MV vector to generate MV-E-NS1(2) by In-Fusion cloning (Stellar cells). PCR amplification of the coding region of Zika prME-NS1 from the gene synthesized plasmid was performed using primers MV-coZprME-NS1(6) Fwd (5′-ACAGAGTGATACGCGTACGGGCCACCATGGGGGCTGATAC-3′) and MV-coZprME-NS1(6) Rev (5′-TCTATTTCACACTAGTGCGATCGCGACGTCGGCTATGCTGTCACC-3′). The PCR fragment was digested and inserted into the ATS-6 MV vector to generate MV-E-NS1(6). PCR amplification of the coding region of Zika prME-NS1 from the gene synthesized plasmid was performed using primers MV-coNS1 Fwd 1 5′- ACAGAGTGATACGCGTACGGGCCACCATGGAGACAGACACACTCCT-3′ and MV-coNS1 Rev 1 5′- GCACGCGATCGCAAGACGTCGGCTATGCTGTCACCATAGAGCGGACCAGGTTG-3′ was used to generate fragment 2. Fragment 2 was digested by restriction enzymes MluI, SgrAI. To insert the human IgKappa signal peptide at ATS-2 to allow for better secretion of ZIKV-NS1, fragment 1 was generated by PCR amplification using primers IgKappa Fwd 2 5′-ACAGAGTGATACGCGTGGCCACCATGGAGACAGACACACTCCTGCTATGGGTACTGCTGCTCTGGGTTCCAGGTTCCACG-3′ and IgKappa Rev 2 5′-ACACGAACACGCCGGTGCCACACCGTGTCTCCTTCTTAGAAAAATCCACGGAGCATCCCACGTCACCAGTGAACCTGGAACCCAGA-3′. The PCR fragments 1 and 2 were inserted into the ATS-2 MV vector to generate MV-NS1(2) by In-Fusion cloning (Stellar cells).

### Virus recovery

In total, 293 T/T17 cells (0.8 × 10^6^ cell per well) were pre-seeded in 6 well plate. The MV-ZIKV viruses and control MV’s were rescued from their full-length cDNA with the helper-plasmid rescue system. 293 T/T17 cells were transfected with pCAGGS-T7, pTIT-MVN (MV-Nucleoprotein), pTIT-MVP (MV-Phosphoprotein), and pEMC-MVLa (MV-Large protein), using the XtremeGene9 reagent (Millipore Sigma). After overnight incubation at 34 °C, the cells were heat-shocked at 42 °C for 3 h and then returned to 34 °C. After 3 days of incubation at 34 °C, transfected cells were transferred onto a monolayer of Vero cells and incubated at 34 °C. Virus was harvested from Vero cells when syncytia involved 80–90% of the culture by scraping infected cells, freeze-thawing cells and medium, and centrifuging them to remove cellular debris.

### Virus production

The Measles viruses were grown in a T175 flask with sub-confluent Vero cells in Optipro SFM. The medium was changed every 3–4 days and the supernatant was collected until the cell monolayer came off. The low passage MV Edmonston strain (ATCC-VR 24) was propagated by inoculating 100 μL of the original ATCC vial into a T175 flask pre-seeded with Vero cells (80% confluent) in 32 mL of 1X DMEM (2% FBS) and placed 34 °C in a humidified 5% CO_2_ atmosphere. Virus supernatant was harvested after 12 days. The virus was harvested by freeze-thawing cells and medium, centrifuging them at 2103 × *g* for 10 min to remove cellular debris. Viral supernatants were tittered, aliquoted, and frozen at –80 °C.

Zika viruses were grown in a T175 flask with sub-confluent Vero cells in 1X DMEM (2% FBS, 1% PS). The medium was changed every 3–4 days, and the supernatant was collected until the cell monolayer came off. The harvested virus was tittered on Vero cells, and high titer stocks were aliquoted and frozen at –80 °C

### Virus titration

#### Measles virus titration

Measles virus titers were analyzed as 50% tissue culture infectious dose (TCID50) by the Reed–Muench method. 10^4^ Vero cells in 100 μL per well were pre-seeded in a 96 well flat-bottom plate 2 h before the addition of virus to the well. In total, 180 μL of 1X DMEM (Corning, Cat# 10-017-CV) was added to every well of the 96 well round bottom plate (dilution plate). In total, 20 μL of one virus was added to Column 1 of the dilution plate. Twelve 10-fold serial dilutions of the virus were performed in the dilution plate in a total volume of 200 μL per well. In total, 30 μL of the diluted virus was transferred from one row to each row of the 96 well plates pre-seeded with cells, changing tips between each row. Two plates were prepared per inoculum. Plates were incubated at 34 °C for 4 days. On day 4, plates were fixed with 80% Acetone for 10 min at 4 °C. Fixation solution was aspirated, and plates were allowed to air dry. Cells were blocked for 1 h in FACS buffer and stained with 100 μL of anti-MV-N cl25 mouse monoclonal antibody (2 μg/mL in FACS buffer-1X PBS, 10%FBS, 0.05% Sodium azide) for 2 h. Plates were washed 3X with FACS buffer and stained with secondary antibody, Cy3-conjugated Goat Anti-mouse IgG (2 μg/mL in FACS buffer) for 2 h. After 3X washes with FACS buffer and plates were read using a fluorescence microscope. The TCID_50_ titer was calculated with the following formula:

$$Log_{10}\left( {TCID_{50}/ml} \right) = L + d\left(\frac{s}{8}--0.5\right) + Log_{10}\left(\frac{1}{v}\right)$$

Where *L* is the reciprocal of the last dilution in which all wells are positive, *d* is the log_10_ of dilution factor, *v* is the volume of inoculum (mL/well).

#### Zika virus titration

ZIKV stocks were propagated in Vero cells or C6/36 cells and titrated by focus-forming assay (FFA)^68^. Briefly, ten-fold serial dilutions of ZIKV in 1X DMEM (Corning, Cat# 10-017-CV) supplemented with 2% fetal bovine serum (R&D systems, Cat# S11150) and 20 U/mL Penicillin-Streptomycin (Gibco, Cat# 1540122) were performed in 96-well Costar (Corning, NY) plates. In total, 2.5 × 10^4^ Vero or C6/36 cells per well were added to the 96 well plate incubated undisturbed for 3 days at 34 °C. Media overlay was aspirated, and cell monolayer was fixed with 80% Acetone for 10 min at 4 °C. Fixation solution was aspirated, and plates were allowed to air dry. Cells were blocked for 1 h in FACS buffer and stained with Pan-Flavivirus E mouse monoclonal (1 μg/mL in FACS buffer) for 2 h. Cells were then washed 3X with FACS buffer and stained with secondary antibody, Cy3-conjugated Goat Anti-mouse IgG (Jackson ImmunoResearch, 2 μg/mL in FACS buffer) for 2 h. After 3X washes with FACS buffer and plates were read using a fluorescence microscope. For each sample, a dilution with easily distinguished foci is selected and titer is calculated in focus-forming units per ml (FFU/ml), using the average of triplicate wells.

$${{{\mathrm{FFU}}}}/{{{\mathrm{ml}}}} = \left( {{{{\mathrm{mean}}}}\;{{{\mathrm{foci}}}}/{{{\mathrm{well}}}}} \right) \times \left( {{{{\mathrm{dilution}}}}\;{{{\mathrm{factor}}}}} \right)/\left( {{{{\mathrm{ml}}}}\;{{{\mathrm{inoculum}}}}} \right)$$

### Multi-step growth curve

We infected Vero-CCL81 cells with wt. or recombinant viruses at a multiplicity of infection (MOI) of 0.001, in triplicate at 34 °C. The infected cells and supernatant were harvested at 12, 24, 48, 72, 96, 120, and 144 hpi respectively. After three cycles of freeze-thawing and sonication of infected cells, lysates were centrifuged to remove cellular debris, and the supernatant was collected. The titer (TCID_50_/ml) of each sample was measured using Vero-CCL81 cells.

### Immunofluorescence assay (IFA)

8.5 × 10^4^ Vero-E6 cells were seeded in 24 well plates with coverslips. After 18 h, the cells were infected with the recovered MV-ZIKV viruses and controls at an MOI of 0.1 for 72 h at 34 °C. The cells were permeabilized for 20 min at room temperature with BD Cytofix/Cytoperm (BD, 554714) and blocked with FACS buffer for 30 min. For Fig. 1b, Cells were stained with Biofront ZIKV-E mouse monoclonal antibody (1 μg/mL) for 1 h at room temperature (RT) on a rocker platform. Cells were washed 3X with FACS buffer and stained with secondary antibody Cy3-conjugated Goat Anti-mouse IgG (Jackson ImmunoResearch, 2 μg/mL in FACS buffer) for 1 h at RT. For Fig. 3b, cells were stained with Biofront ZIKV-E mouse monoclonal antibody (1 μg/mL) and Anti-ZIKV-NS1 human monoclonal antibody EB9 (2μg/mL) for 1 h at room temperature (RT) on a rocker platform. Cells were washed 3X with FACS buffer and stained with secondary antibodies Alexa Fluor 568 -conjugated goat anti-mouse IgG (ThermoFisher Scientific, 2.5 μg/mL), and Alexa Fluor 647 conjugated goat α-human IgG (ThermoFisher Scientific, 2.5 μg/mL), for 1 h at RT. For both Figs. 1b and 3b, cells were washed 3X with FACS buffer and stained with anti-MV-N cl25 mouse monoclonal antibody conjugated with Dylight 488 (5 μg /mL) at RT for 1 h. Cells were washed 3X with FACS buffer and mounted with VECTASHIELD® Hardset™ Antifade Mounting Medium with DAPI (H-1500). Images were taken using Nikon A1R + confocal microscope.

### Viral sucrose purification and cell lysates

Larger amounts of MV-ZIKV and control MV supernatants were spun through a 20% sucrose cushion in an SW32 Ti rotor (Beckman, Inc.) at 106,559 × *g* for 2 h. ZIKV was spun through a 20% sucrose cushion at 153,446 xg for 3.5 h. Virion pellets were resuspended in phosphate-buffered saline (PBS), and protein concentrations were determined using a bicinchoninic acid (BCA) assay kit (Pierce). 6 well plates seeded with 0.7 ×106 Vero cells, 16 h before they were infected with MV-ZIKV viruses and control viruses at an MOI of 5 at 34 °C for 60 h and harvested using Sabatini Buffer (40 mM Tris,ph7.6; 120 mM NaCl; 1 mM TRITON-X100; 0.4 mM Sodium Deoxycholate; 1 mM EDTA), and protein concentrations were determined using a bicinchoninic acid (BCA) assay kit (Pierce). The uncropped images of Figs. 1c–e, 3c–e, and Supplementary Fig. 5a-b are provided in Supplementary Figs. 7, 8, 9 respectively.

### Purification of subviral particles (SVPs)

T175 flasks were infected with MV-ZIKV vaccines or controls at an MOI of 0.1 at 34 °C. Larger amounts of MV-ZIKV and control MV and Zika virus supernatants were filtered through a 0.2 μm filter (Rapid-Flow™ Sterile Disposable Bottle Top Filters with PES Membrane). The 0.2 μm filtration step was aimed to filter most of the MV particles (pleomorphic, 100–300 nm) out of the supernatant. The filtered supernatant was then spun through a 20% sucrose cushion at 279,232.1 × *g* for 3 h in a SW55Ti rotor (Beckman Coulter). The SVP pellets were resuspended in a 4X non-reducing buffer (Alfa Aesar, ThermoFisher, Cat# J63615-AD) in a total volume of 100 μL. SVPs for MV-E0 virus could not be purified and were therefore not included in this blot.

### SDS-PAGE and western blot

The sucrose purified virus particles or cell lysates were denatured with 4X Laemmli Sample Buffer supplemented with 2-mercaptoethanol (10%) at 95°C for 10 min. In total, 2.5 μg of sucrose purified virus or 5 μg of cell lysates or 10 μL of the SVPs (in non-reducing buffer) was resolved on a 10% SDS–polyacrylamide gel and thereafter stained overnight with SYPRO Ruby for total protein analysis or transferred onto a nitrocellulose membrane in Towbin buffer (192 mM glycine, 25 mm Tris, 20% methanol) for Western blot analysis. The nitrocellulose membrane was then blocked in PBST (1X PBS, 0.05% Tween-20) containing 5% dried milk at room temperature for 1 h. After blocking, the membrane was washed 3X with PBST and incubated overnight with Biofront ZIKV-E mouse monoclonal antibody (1 μg/mL) or anti-MV-H Rabbit polyclonal sera (diluted 1:5000) or anti-MV-N cl25 mouse monoclonal antibody (1 μg/mL) or anti-ZIKV-NS1 B4 mouse monoclonal antibody (Abcam,1μg/mL) in 10% bovine serum albumin (BSA). After washing, the blot was incubated for 1 h with horseradish peroxidase (HRP)-conjugated anti-mouse/human/rabbit IgG diluted 1:20,000 in blocking buffer depending on the primary antibody used. Bands were developed with SuperSignal West Dura Extended duration substrate (Pierce).

### ZIKV envelope antigen

Recombinant Zika Envelope protein antigen: The antigen used for ELISA and ELISPOT assay was purchased from Aalto BioRegents (AZ 6312).

### Recombinant Measles virus H and ZIKV-NS1 antigen

A codon optimized MV-H gene (Edmonston B strain) was gene synthesized by GenScript. PCR amplification of the coding region of MV-H from the plasmid was performed using primers coMV-H61 N HA FWD (5′-TCGTGGTGCCAGATCTCACAGAGCCGCCATCTAT - 3′) and coMV-H61 N-HA REV (5′- TCTCGAGCGGCGGCCGCCTACCTTCTATTTGTGCCG -3′) to generate a fragment from amino acid 61 to 617 of MV-H protein. The PCR amplified fragment was inserted by In-Fusion cloning (Stellar cells) into the pDisplay vector containing N terminal hemagglutinin (HA) tag that was cut with restriction enzymes BglII and NotI.

A codon-optimized Zika prME-NS1 gene (strain PRVABC59 Asian 2015; GenBank: ANW07476.1) was gene synthesized by GenScript. The recombinant ZIKV-NS1 was constructed as published previously^13^. Briefly, PCR amplification of the coding region of ZIKV-NS1 from the plasmid was performed using primers Sol coNS1 Fwd 5′- TGACGCACCTAGATCTAATGGCCCATCTCTCTGATGTGC - 3′ and Sol coNS1 Rev1 5′- CGTATGGATAGTCGACAGCACGTCCTGCTGTCACCATAGAGCGGACC-3′ to generate a fragment that incorporated the last 24 amino acids of ZIKV envelope (NGSISLMCLALGGVLIFLSTAVSA) to the amino terminus of the NS1 coding region (Amino acid 1-352). The PCR amplified fragment was inserted by In-Fusion cloning (Stellar cells) into the pDisplay vector containing C terminal HA tag that was cut with restriction enzymes BglII and SalI.

Sub-confluent T175 flasks of 293 T cells (human kidney cell line) were transfected with XtremeGene9 (150 μL/flask) and a pDisplay vector encoding either the codon-optimized MV-H fused to an N-terminal HA tag (50μg/flask) or codon-optimized ZIKV-NS1 fused to a C-terminal HA tag (50μg/flask) at 37 °C. Supernatant was collected between days 7 post-transfection and loaded onto two different equilibrated anti-HA agarose (Pierce) columns containing a 2.5-ml agarose bed volume. The supernatant is recirculated overnight at 4 °C using a peristaltic pump at 1 ml/minute. The column was washed with 10-bed volumes of PBS (0.05% Sodium Azide). After washing, antibody-bound MV-H was eluted with 5 ml of 250 μg/ml HA peptide in PBS. Fractions were collected and analyzed for MV-H by Western blotting with monoclonal anti-HA-7 antibody (Sigma) prepared in 10% BSA. Peak fractions were then pooled and dialyzed against PBS in 10,000 molecular weight cutoff (MWCO) dialysis cassettes (Thermo Scientific) to remove excess HA peptide. After dialysis, the protein was quantitated by UV spectrophotometry and frozen in small aliquots at −80 °C.

### Enzyme-linked immunosorbent assay

We tested individual mouse sera by enzyme-linked immunosorbent assay (ELISA) for the presence of IgG specific to ZIKV-E, ZIKV-NS1 and MV-H. To test for anti-ZIKV-E humoral responses, recombinant ZIKV-E (Alto BioRegents) was resuspended in coating buffer (50 mM Na2CO3 [pH 9.6]) at a concentration of 1 μg /ml and then plated in 96-well ELISA MaxiSorp plates (Nunc) at 100 μl in each well. ZIKV-NS1 and MV-H were similarly resuspended in coating buffer (50 mM Na2CO3 [pH 9.6]) at a concentration of 1 μg/ml and then plated in 96-well ELISA MaxiSorp plates (Nunc) at 100 μl per well. After overnight incubation at 4 °C, plates were washed three times with 1X PBST (0.05% Tween 20 in 1× PBS), which was followed by the addition of 250 μl blocking buffer (5% dry milk powder in 1× PBST) and incubation at room temperature for 1 h. The plates were then washed three times with PBST and incubated overnight at 4°C with serial dilutions of sera in 1X PBST containing 0.5% BSA, 0.05% Sodium azide. Plates were washed three times the next day, followed by the addition of horseradish peroxidase-conjugated goat anti-mouse-IgG Fc secondary antibody (1:2000) (Southern Biotech, 1033-05). After incubation for 2 h at room temperature, plates were washed three times with PBST, and 200 μl of o-phenylenediamine dihydrochloride (OPD) substrate (Sigma) was added to each well. The reaction was stopped by the addition of 50 μl of 3 M H_2_SO_4_ per well. Optical density was measured at 490 nm (OD490) using BioTek Spectrophotometer. ELISA data were analyzed with GraphPad Prism 8. using a sigmoidal nonlinear fit model to determine the 50% effective concentration [EC50] titer. The EC50 titer is the concentration (dilution) at which the antibody/serum at which you get 50% of your maximal effect (Optical Density).

### Zika neutralization assay

A FRNT measured ZIKV neutralizing antibody assay was performed^69^. Briefly, heat-inactivated (56°C, 30 min) sera were serially diluted (three-fold) starting at a 1/30 dilution and incubated with 10^2^ FFU of ZIKV (strain /PRVABC59/2015/P1 Vero) for 1 h at 34°C. The ZIKV-serum mixtures were added to Vero cell monolayers in 96-well plates (1.2 × 10^4^ Vero cells per well were seeded 16 h prior to virus addition) and incubated for 1.5 h at 34°C, followed by overlaying the cells with 1% (w/v) methylcellulose in 1X DMEM (5%FBS). Cells were incubated for 40 h at 34 °C and subsequently fixed using 2% PFA in PBS for 1 h at room temperature. Cells were permeabilized with Perm buffer (1X PBS, 5% FBS, 0.2% Triton X-100) for 20 min at 4 °C and washed 3X with FACS buffer (1XPBS, 5% FBS, 0.05% Sodium azide). ZIKV-infected cell foci were detected using anti-Flavivirus E 4G2 mouse monoclonal antibody (1ug/mL), washed 3X with FACS buffer, followed by Cy3-conjugated Goat Anti-mouse IgG (Jackson ImmunoResearch, 2 μg/mL). After 3X washes with FACS buffer and plates were read using a fluorescence microscope. For Figs. 4, 5, and 6, the 1st International Standard for anti-Asian lineage Zika virus antibody (NIBSC: 16/352) and Working reagent for anti-Zika virus antibody (NIBSC: 16/320) were used at a starting dilution of 1:100. The 50% reduction point (RP -Reciprocal of the dilution where 50% neutralization is observed) for the serum and standards were noted and the FRNT_50_ titer was calculated as follows:

$$Antibody\;titre\left( {IU/ml} \right) = (antibody\;titre\;of\;standard\;serum) \times \left(\frac{{50\% \;RP\;of\;test\;serum}}{{50\% RP\;of\;standard\;serum}}\right)$$

### Measles neutralization assay

Measles neutralization assay was performed on heat-inactivated sera^70^. Sera and the 3rd International standard for Anti-Measles serum were heat-inactivated at 56 °C for 30 min. In a 96 well plate serum samples were diluted serially 4-fold from 1/10 and the 3rd International standard was diluted 1/100 in 1X DMEM (2% FBS, 1%PS) 70 μl and mixed with 30 μl volume of diluted virus solution (150 PFU/well) of low-passage MV Edmonston strain (P1, Vero) in 1X DMEM (2% FBS, 1%PS) on a plate shaker at 34°C for 1 h. Then, 1.2 × 10^4^ Vero cell suspension was added (100 μl) and incubated for 68 h at 34 °C. Cells were fixed with 80% acetone for 10 min at 4 °C. Fixation solution was aspirated, and plates were allowed to air dry. Cells were blocked for 1 h in FACS buffer and stained with 100μL of anti-MV-N cl25 mouse monoclonal antibody (2 μg/mL in FACS buffer-1X PBS, 10%FBS, 0.05% sodium azide) for 2 h. Plates were washed 3X with FACS buffer and stained with secondary antibody, Cy3-conjugated Goat Anti-mouse IgG (2 μg/mL in FACS buffer) for 2 h. After 3X washes with FACS buffer and plates were read using a fluorescence microscope the presence of measles virus was detected by direct EIA as described above. All serum dilutions were tested in triplicate. The 50% reduction point (50%RP) of each serum was calculated using the Reed–Muench formula. The neutralizing antibody titer of test sera was converted into mIU/ml by comparing their 50% RP with that of the international standard serum using the following formula:

$$Antibody\;titre\left( {mIU/ml} \right) = (antibody\;titre\;of\;standard\;serum) \times \left(\frac{{50\% \;RP\;of\;test\;serum}}{{50\% RP\;of\;standard\;serum}}\right)$$

### RNA extraction

Whole blood (50 µL) was resuspended in 150 µL of TRIzol LS Reagent (Life Technologies). All Organs were added to Omni pre-filled bead tubes containing 1 mL of TRIzol and homogenized using the OMNI bead ruptor 12. The RNA extraction protocol for biological fluids using TRIzol LS Reagent was followed until the phase separation step. The remaining RNA extraction was done using the PureLink RNA Mini Kit (Ambion). The quantity and quality (260/280 ratios) of RNA extracted was measured using NanoDrop (Fisher).

### Quantification of Zika virus RNA by quantitative Real-Time polymerase chain reaction

ZIKV cDNA was generated from RNA isolated from the ZIKV African MR766 strain and Asian PRVABC59 strain by One-Step RT PCR (SuperScript III, Thermo Fisher Scientific) with primers ZKV NS4B IVT F1 5′-GAATTCTAATACGACTCACTATAGGGGCATCTAATGGGAAGGAGA-3′ and ZKV NS4B IVT R1 (5′-GCTAGCGGCTGTAGAGGAGTTCCAGTA-3′). The African and Asian standards were generated by in-vitro transcription of the generated ZIKV cDNA, followed by using the MEGAclear Transcription Clean-Up Kit. Aliquots of 2 × 10^10^ copies/µL were frozen at –80 °C. Five microliters of RNA per sample were run in triplicate, using the ZIKV-F2 (5′-CAGCTGGCATCATGAAGAATC-3′) and ZIKV-R1 5′-CACTTGTCCCATCTTCTTCTCC-3′ primers for African strain detection (ThermoFisher Scientific) or the ZIKV-F1 (5′-CAGCTGGCATCATGAAGAACC-3′) and ZIKV-R2 5′-CACCTGTCCCATCTTTTTCTCC-3′ primers for Asian strain detection. The panZika-Probe 6FAMGTTGTGGATGGAATAGTGGMGBFNQ detects both the Asian and the African strain. In total, 5 μL of RNA per sample was assayed in triplicate in a one-step RT-qPCR reaction in a total volume of 20 μL. The reaction was set up for a fast cycling mode with the following thermal cycling parameters: 1cycle for 5 min at 50 °C, 1cycle for 20 s at 95 °C, 40 cycles of 95 °C for 3 s and 60 °C for 30 s. For all assays except Fig. 6, Taqman Fast virus 1-Step master mix (ThermoFisher) was used. For Fig. 6, Taqpath 1 step RT-qPCR mix (ThermoFisher) was used. The thermal cycling parameters for Taqpath master mix: 1 cycle for 2 min at 25 °C, 1cycle for 15 min at 50 °C, 1 cycle for 2 min at 95 °C, 40 cycles of 95 °C for 3 s, and 60 °C for 30 s. The reactions were run on Step One Plus qPCR machine.

### ELISPOT for assessing plasma cells

An ELISPOT assay quantitated the number of ZIKV E, ZIKV-NS1 and MV-H specific plasma cells in the bone marrow and spleen respectively^71^. ELISPOT plates (Millipore) were coated with ZIKV-E antigen (50 μg/mL), ZIKV-NS1 antigen (50 μg/mL) and MV-H antigen (10 μg/mL), overnight at 4 °C. Subsequently, plates were washed six times with PBS (200 μl) and then blocked for 1–2 h with Goat Serum (ThermoFisher Scientific, 16210072) at 37 °C. Bone marrow cells from the femurs and splenocytes were harvested from the immunized mice and controls, and erythrocytes were lysed by ACK lysis buffer. Subsequently, 3 × 10^6^ cells/well were added to the ZIKV-E and ZIKV-NS1 coated plates, and 1 × 10^6^ cells/well were added to the MV-H coated plates. Cells were serially diluted in a 96 well round bottom plate, transferred to the coated ELISPOT plate, and incubated overnight at 37 °C in a CO_2_ incubator for 16 h. Plates were washed four times with 1X PBST, incubated with HRP conjugated goat anti-mouse IgG-Fc (Sothern Biotech, 1μg/mL) in PBS-T for 2 h at 37 °C. Following 3X washing with 1X PBST, plates were washed 3X with PBS. Spots were developed with TrueBlue peroxidase substrate (KPL) before the reaction was quenched with water and counted with an AID EliSpot Reader (Autoimmun Diagnostika GmbH).

### Antibody-dependent effector functions

For experiments involving infected cells, Vero cells were seeded on 96-well, flat, white-bottom plates (Corning) and infected after 48 h with Zika virus PRVABC59 at an MOI of 0.01 at 34°C. After 48 h, the medium was removed and 25 μl of assay buffer (RPMI 1640 with 4% low-IgG FBS) was added to each well. Sera was pooled from each group and heat inactivated for analysis. Then, sera were added in a volume of 25 μl at a starting dilution of 1:10 and serially diluted threefold in assay buffer in duplicate. The sera were then incubated with the infected cells for 30 min at 34 °C. Anti-Zika virus NS1 EB9 human monoclonal antibody was used as a control with human Jurkat cells. Genetically modified Jurkat cells expressing either mouse FcγR IV or human FcγR IIIa with a luciferase reporter gene under the transcriptional control of nuclear-factor-activated T cell (NFAT) promoter were added at 1.5 × 10^5^ cells in 25 μl per well, which is approximately a 1:5 ratio of target cells to effector cells (Promega). Cells were then incubated for another 6 h at 34 °C. After the incubation, 75 μL Bio-Glo Luciferase assay reagent was added, and luminescence was quantified using a microplate reader. Fold induction was measured in relative light units and calculated by subtracting the background signal from wells without effector cells and then dividing values for wells with antibody by values for those with no antibody added. Specifically, fold induction was calculated as follows: *(RLU induced – RLU background)/(RLU uninduced – RLU background)*. The mean values and standard errors of the means (SEM) were reported, and a nonlinear regression curve was generated using GraphPad Prism 9.

### Fetal brain histology

Fetal brains were harvested in 4% PFA and stored at 4 °C. The tissue was dehydrated, fixed and infiltrated with paraffin on the Leica Polaris 2 tissue processor. The infiltrated tissue was embedded and sectioned at 5 microns. Antigen retrieval was performed on the Roche Ventana Discovery ULTRA staining platform using Discovery CC1 (Roche cat#950-500) for a total application time of 64 min. The primary antibody, human anti-Zika-NS1(EB9) (2 mg/mL), was incubated as a 1:400 dilution, at room temperature, for 44 min. Secondary immunostaining used a horseradish peroxidase (HRP) multimer cocktail (Roche cat#760-500) and immune complexes were visualized using the ultraView Universal DAB (diaminobenzidine tetrahydrochloride) Detection Kit (Roche cat#760-500). Slides were then washed with a Tris based reaction buffer (Roche cat#950-300) and counter-stained with Hematoxylin II (Roche cat #790-2208) for 4 min.

### Statistical analysis

Specific statistical tests used to analyze experimental datasets are described in the respective Figure Legends. For experiments with only female mice, antibody responses and immune cell analyses, One-way ANOVA with post-hoc Tukey HSD test was performed on log transformed data for each time point. For data analysis where only two groups were compared, a Mann–Whitney U test was performed on log transformed data for each time point. For experiments with female and male mice, antibody responses, and immune cell analyses, two-way ANOVA with post-hoc Tukey HSD test was performed on log transformed data for each time point. Survival curves were analyzed using the log rank test with a Bonferroni correction. For Fig. 6, we performed a Pairwise Two-Sided Multiple Comparison Analysis of log transformed data by the Dwass, Steel, Critchlow-Fligner method. A P value of <0.05 was assigned to establish statistical significance using GraphPad Prism version 9.0.

### Reporting summary

Further information on research design is available in the Nature Research Reporting Summary linked to this article.

## Supplementary information

REPORTING SUMMARY

Supp Info

## Data Availability

All data generated from this study are present in the paper or Supplementary Materials. The datasets generated during and/or analyzed during the current study are available from the corresponding author on reasonable request.

## Materials availability

Vaccines and Reagents can be shared with a standard material transfer agreement upon request to the corresponding author, Matthias J Schnell
