# Supplementary material for: Measles-based Zika vaccine induces long-term immunity and requires NS1 antibodies to protect the female reproductive tract
Source: NPJ Vaccines. 2022 Apr 19;7:43. doi: 10.1038/s41541-022-00464-2 (PMC9018676; doi:10.1038/s41541-022-00464-2)
Supplement: Supplementary file 1 — REPORTING SUMMARY [file 41541_2022_464_MOESM1_ESM.pdf]

nature portfolio

Corresponding author(s): Matthais J. Schnell

Last updated by author(s): Feb 7, 2022

# Reporting Summary

Nature Portfolio wishes to improve the reproducibility of the work that we publish. This form provides structure for consistency and transparency in reporting. For further information on Nature Portfolio policies, see our Editorial Policies and the Editorial Policy Checklist.

## Statistics

For all statistical analyses, confirm that the following items are present in the figure legend, table legend, main text, or Methods section.

| n/a | Confirmed | |
|---|---|---|
| ☐ | ☒ | The exact sample size ($n$) for each experimental group/condition, given as a discrete number and unit of measurement |
| ☐ | ☒ | A statement on whether measurements were taken from distinct samples or whether the same sample was measured repeatedly |
| ☐ | ☒ | The statistical test(s) used AND whether they are one- or two-sided<br>*Only common tests should be described solely by name; describe more complex techniques in the Methods section.* |
| ☐ | ☒ | A description of all covariates tested |
| ☐ | ☒ | A description of any assumptions or corrections, such as tests of normality and adjustment for multiple comparisons |
| ☐ | ☒ | A full description of the statistical parameters including central tendency (e.g. means) or other basic estimates (e.g. regression coefficient) AND variation (e.g. standard deviation) or associated estimates of uncertainty (e.g. confidence intervals) |
| ☒ | ☐ | For null hypothesis testing, the test statistic (e.g. $F$, $t$, $r$) with confidence intervals, effect sizes, degrees of freedom and $P$ value noted<br>*Give P values as exact values whenever suitable.* |
| ☒ | ☐ | For Bayesian analysis, information on the choice of priors and Markov chain Monte Carlo settings |
| ☒ | ☐ | For hierarchical and complex designs, identification of the appropriate level for tests and full reporting of outcomes |
| ☒ | ☐ | Estimates of effect sizes (e.g. Cohen's $d$, Pearson's $r$), indicating how they were calculated |

*Our web collection on statistics for biologists contains articles on many of the points above.*

## Software and code

Policy information about availability of computer code

| | |
|---|---|
| Data collection | Microsoft Excel |
| Data analysis | PRISM software, Aperio Imagescope |

For manuscripts utilizing custom algorithms or software that are central to the research but not yet described in published literature, software must be made available to editors and reviewers. We strongly encourage code deposition in a community repository (e.g. GitHub). See the Nature Portfolio guidelines for submitting code & software for further information.

## Data

Policy information about availability of data

All manuscripts must include a data availability statement. This statement should provide the following information, where applicable:

- Accession codes, unique identifiers, or web links for publicly available datasets
- A description of any restrictions on data availability
- For clinical datasets or third party data, please ensure that the statement adheres to our policy

All data generated from this study are present in the paper or Supplementary Materials. The datasets generated during and/or analyzed during the current study are available from the corresponding author on reasonable request.

# Field-specific reporting

Please select the one below that is the best fit for your research. If you are not sure, read the appropriate sections before making your selection.

☒ Life sciences ☐ Behavioural & social sciences ☐ Ecological, evolutionary & environmental sciences

For a reference copy of the document with all sections, see nature.com/documents/nr-reporting-summary-flat.pdf

# Life sciences study design

All studies must disclose on these points even when the disclosure is negative.

| | |
|---|---|
| Sample size | Each animal study performed with 5 0r 10 animals per group |
| Data exclusions | No data was excluded |
| Replication | Each assay was done in replicates of 2 or 3 and repeated as per the N mentioned in each figure legend |
| Randomization | Animals were randomly assigned to a group |
| Blinding | Blinding was not possible |

# Reporting for specific materials, systems and methods

We require information from authors about some types of materials, experimental systems and methods used in many studies. Here, indicate whether each material, system or method listed is relevant to your study. If you are not sure if a list item applies to your research, read the appropriate section before selecting a response.

## Materials & experimental systems

| n/a | Involved in the study | |
|---|---|---|
| ☐ | ☒ | Antibodies |
| ☐ | ☒ | Eukaryotic cell lines |
| ☒ | ☐ | Palaeontology and archaeology |
| ☐ | ☒ | Animals and other organisms |
| ☒ | ☐ | Human research participants |
| ☒ | ☐ | Clinical data |
| ☒ | ☐ | Dual use research of concern |

## Methods

| n/a | Involved in the study | |
|---|---|---|
| ☒ | ☐ | ChIP-seq |
| ☒ | ☐ | Flow cytometry |
| ☒ | ☐ | MRI-based neuroimaging |

## Antibodies

| | |
|---|---|
| Antibodies used | The following antibodies were used in this study: Anti-ZIKV-E mouse monoclonal antibody (Biofront Technologies, 1176-56), Pan-Flavivirus-E 4G2 mouse monoclonal antibody produced from hybridoma cell line D1-4G2-4-15 (ATCC, HB-112), Anti-Measles Nucleoprotein mouse monoclonal antibody produced from hybridoma NP.cl25 (Millipore Sigma, Cat # 95051114), Anti Measles H polyclonal Rabbit sera from Dr. R.Cattaneo, Anti-ZIKV-NS1 human monoclonal antibody EB9 (2[illegible]g/mL) from Dr. Gene Tan. |
| Validation | ZIKV-E ,Biofront, https://www.biofronttech.com/product/life-science-zika-virus-reagents-zika-virus-envelope-monoclonals/anti-zika-envelope-mab-1176-56-500ug/1607012/<br>Pan-Flavivirus-E 4G2 mouse monoclonal antibody produced from hybridoma cell line D1-4G2-4-15 (ATCC, HB-112), https://www.atcc.org/products/vr-1852<br>Millipore Sigma, Cat # 95051114, https://www.sigmaaldrich.com/US/en/product/sigma/cb_95051114<br>Anti Measles H polyclonal Rabbit sera provided and verified by Dr. R.Cattaneo<br>Anti-ZIKV-NS1 human monoclonal antibody EB9 (2mg/mL) provided and verified by Dr. Gene Tan. https://www.nature.com/articles/s41467-018-07008-0 |

## Eukaryotic cell lines

Policy information about cell lines

| | |
|---|---|
| Cell line source(s) | Vero-CCL81, Vero-E6, and 293T/T17 cells were purchased from ATCC, mouse FcγR IV or human FcγR IIIa effector cells were purchased from Promega |
| Authentication | Cells were verified by ATCC and Promega |

| | |
|---|---|
| Mycoplasma contamination | Cells lines were not tested for mycoplasma contamination |
| Commonly misidentified lines (See ICLAC register) | N/A |

## Animals and other organisms

Policy information about studies involving animals; ARRIVE guidelines recommended for reporting animal research

| | |
|---|---|
| Laboratory animals | Mouse, Human CD46-IFNAR-/- mice, male and female from ages of 7-12 weeks were used in this study |
| Wild animals | N/A |
| Field-collected samples | N/A |
| Ethics oversight | Institutional Animal Care and Use Committee at Thomas Jefferson University |

Note that full information on the approval of the study protocol must also be provided in the manuscript.
