# Supplementary material for: Measles-based Zika vaccine induces long-term immunity and requires NS1 antibodies to protect the female reproductive tract
Source: NPJ Vaccines. 2022 Apr 19;7:43. doi: 10.1038/s41541-022-00464-2 (PMC9018676; doi:10.1038/s41541-022-00464-2)
Supplement: Supplementary file 2 — Supp Info [file 41541_2022_464_MOESM2_ESM.pdf]

Supplementary Materials

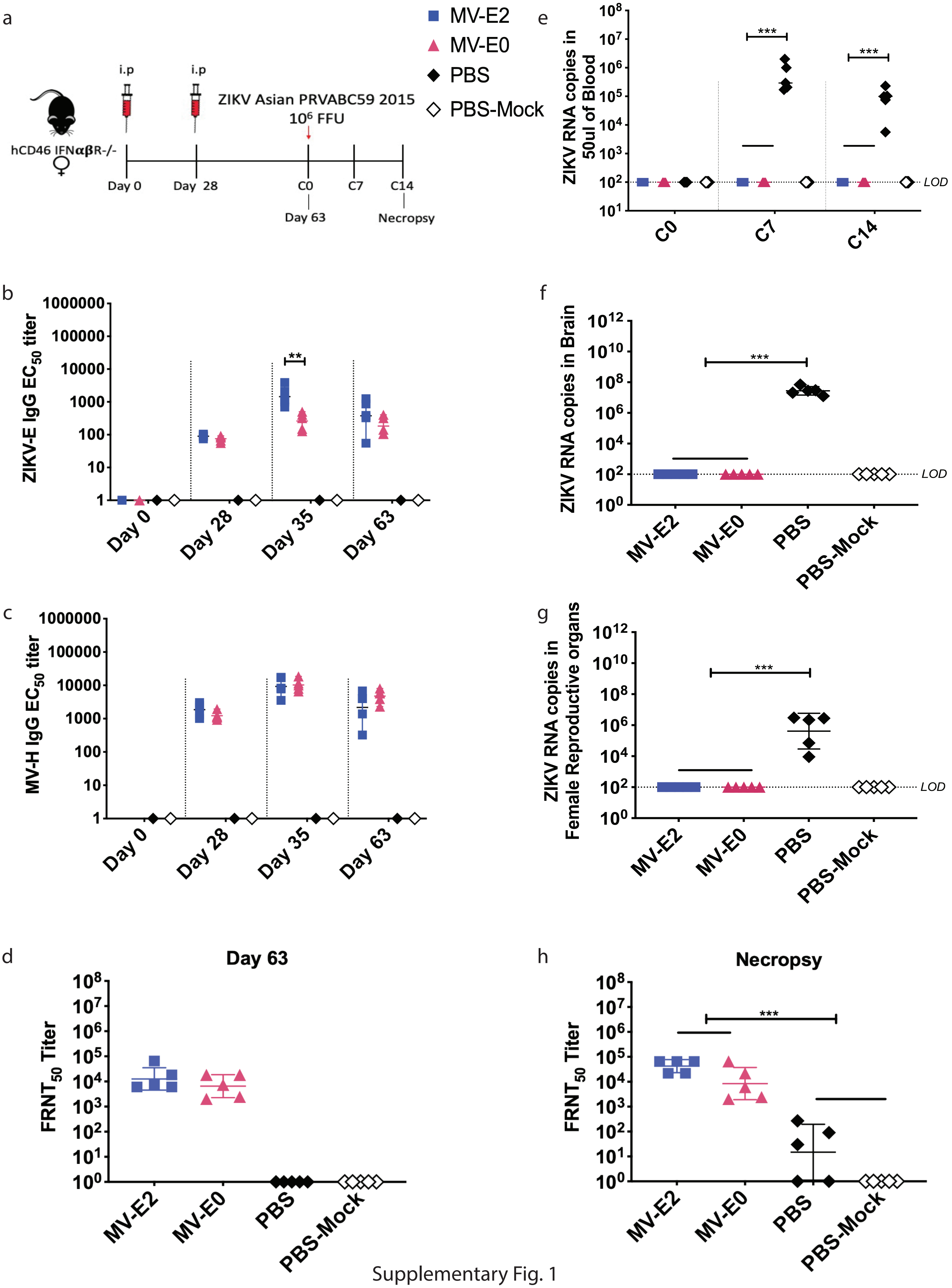

Supplementary Fig. 1

Supplementary Fig. 1. Immunogenicity and efficacy testing of first-generation candidate MV-ZIKV vaccines using a non-lethal ZIKV Asian PRVABC59 challenge strain.

(a) Timeline of vaccination, challenge, and viral load determinations.

(b-c) Anti-ZIKV-E (b) and anti-MV-H (c) specific ELISA IgG EC_{50} titers of the vaccinated animals are plotted on a graph for all animals at different time points. The mean of triplicate EC50 values is depicted per animal. Mean ± SD is depicted per group.

(d & h) ZIKV neutralization with PRVABC59 Asian strain. FRNT assay was performed on day 63 (d) and necropsy (h) sera from vaccinated animals and controls. The mean 50% neutralizing titer ($FRNT_{50}$) of triplicates is plotted for each animal on the graph. The Mean ± SD is depicted per group.

(e-g) ZIKV RNA copies by qPCR in the blood (e), brain (f), and reproductive tract (g). The mean of triplicate RNA copies is depicted per animal. The Mean ± SD is depicted per group. The LOD is 100 copies.

Statistics for Supplementary Fig. 1b-c were done using the Mann-Whitney U test and performed on log-transformed data for each time point. Statistics for Supplementary Fig. 1d-h were done using the one-way ANOVA with post hoc Tukey HSD test and performed on log-transformed data for each time point. Only significant differences are depicted. P-value of 0.12(ns), 0.033(*), 0.002(**), <0.001(***) are depicted accordingly. LOD stands for limit of detection. A horizontal line (——) is used to include all groups below it.

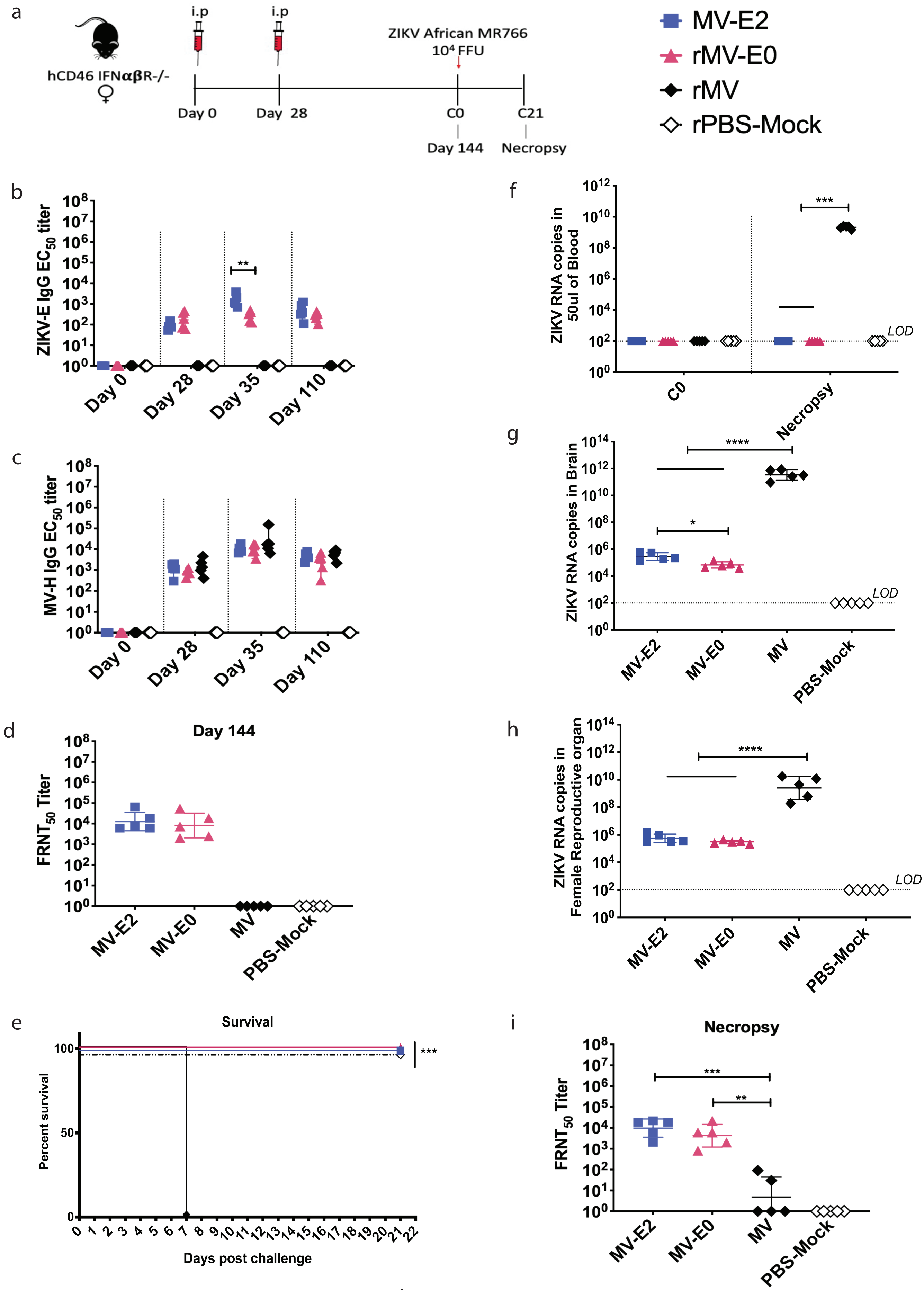

Supplementary Fig. 2

Supplementary Fig. 2. Long-term immunogenicity and efficacy testing of first-generation candidate MV-ZIKV vaccines using a lethal ZIKV African MR766 challenge strain.

(a) Timeline of vaccination, challenge, and viral load determinations.

(b-c) Anti-ZIKV-E (b) and anti-MV-H (c) specific ELISA IgG EC_{50} titers of the vaccinated animals are plotted on a graph for all animals at different time points. The mean of triplicate EC50 values is depicted per animal. Mean $\pm$ SD is depicted per group.

(d & i) FRNT assay was performed on day 144 (d) and necropsy (i) sera from vaccinated animals and controls. The mean 50% neutralizing titer ($FRNT_{50}$) of triplicates is plotted for each animal on the graph. The Mean $\pm$ SD is depicted per group.

(e) Kaplan-Meier survival curve analysis of vaccinated and control animals post-challenge.

(f-h) ZIKV RNA copies by quantitative polymerase chain reaction (qPCR) in the blood (f), brain (g), and reproductive tract (h). The mean of triplicate RNA copies is depicted per animal. The Mean $\pm$ SD is depicted per group. The LOD is 100 copies.

Statistics for Supplementary Fig. 2b-d & f-i was done using the one-way ANOVA with post hoc Tukey HSD test and performed on log-transformed data for each time point. Supplementary Fig. 2e survival curves were analyzed using the log-rank test with a Bonferroni correction. Only significant differences are depicted. P-value of 0.1234(ns), 0.0332(*), 0.0021(**), 0.0002(***), <0.0001(****) are depicted accordingly. A horizontal line (——) is used to include all groups below it.

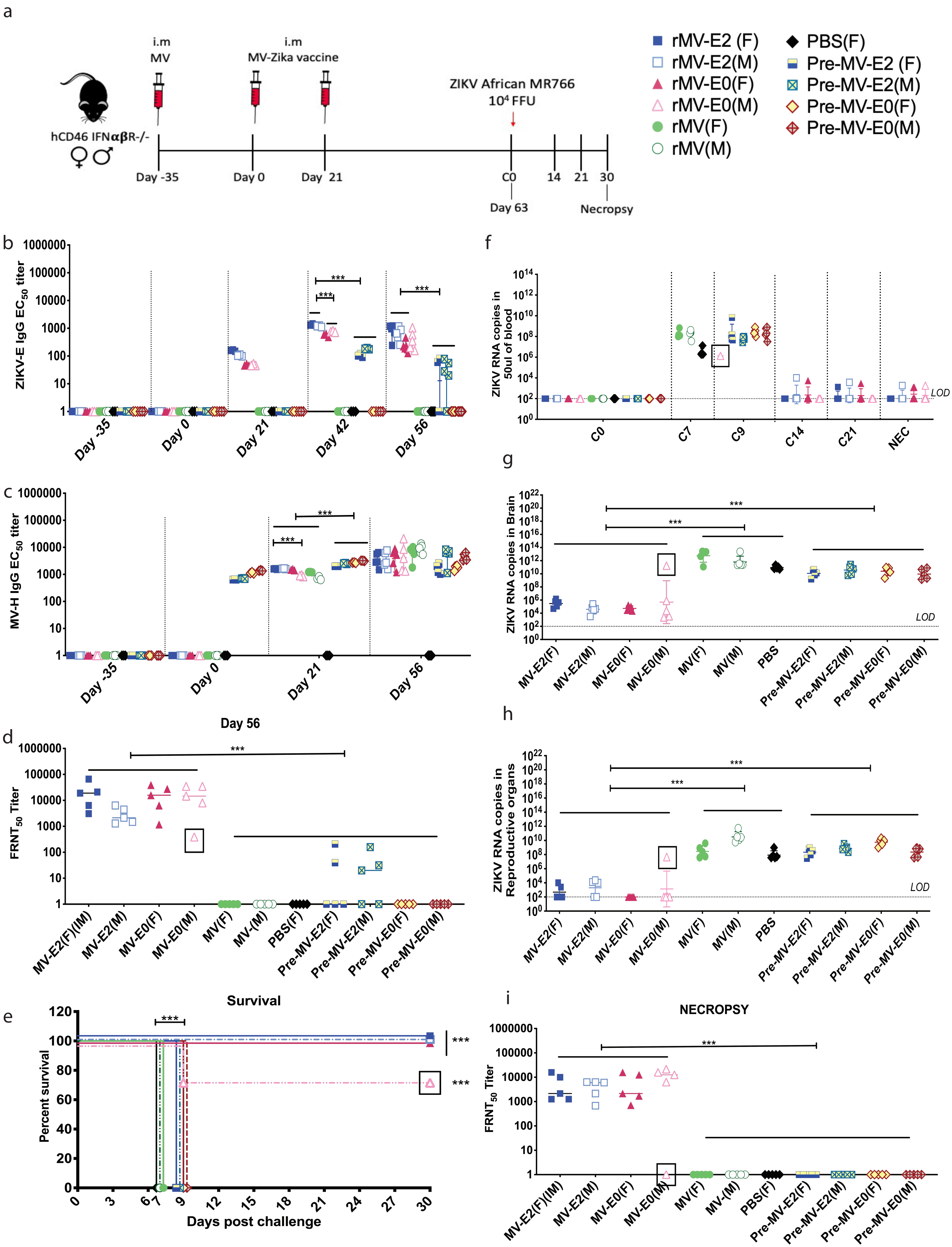

Supplementary Fig. 3

Supplementary Fig. 3. Effect of intramuscular route of vaccination and prior MV immunity to the immunogenicity and efficacy of first-generation candidate MV-ZIKV vaccines using a lethal ZIKV African MR766 challenge strain.

(a) Timeline of vaccination, challenge, and viral load determinations. The black boxed open pink triangle △ is the MV-E0(M) animal that succumbed to ZIKV disease.

(b-c) Anti-ZIKV-E (b) and anti-MV-H (c) specific ELISA IgG EC_{50} titers of the vaccinated animals are plotted on a graph for all animals at different time points. The mean of triplicate EC50 values is depicted per animal. Mean ± SD is depicted per group.

(d & i) FRNT assay was performed on day 63 (d) and necropsy (i) sera. The mean 50% neutralizing titer ($FRNT_{50}$) of triplicates is plotted for each animal on the graph. The Mean ± SD is depicted per group.

(e) Kaplan-Meier survival curve analysis of vaccinated and control animals post-challenge.

(f-h) ZIKV RNA copies by qPCR in the blood (f), brain (g), and reproductive tract (h) at necropsy. The mean of triplicate RNA copies is depicted per animal. The Mean ± SD is depicted per group. The LOD is 100 copies.

Statistics for Supplementary Fig. 3b-d & f-i was done using the two-way ANOVA with post hoc Tukey HSD test and performed on log-transformed data for each time point. Supplementary Fig. 3e survival curves were analyzed using the log-rank test with a Bonferroni correction. Only significant differences are depicted. P-value of 0.1234(ns), 0.0332(*), 0.0021(**), 0.0002(***), <0.0001(****) are depicted accordingly. A horizontal line (——) is used to include all groups below it.

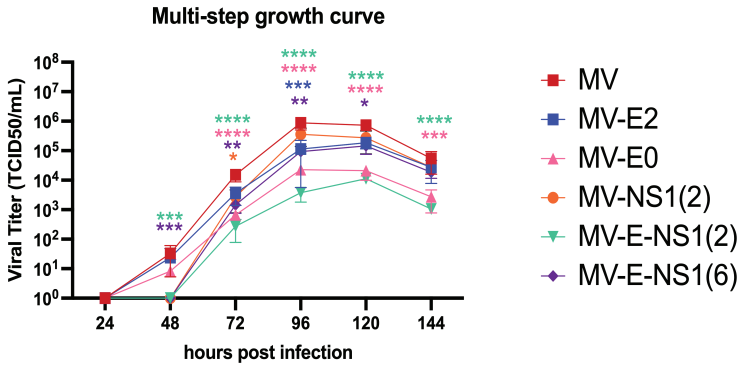

Supplementary Fig. 4

Supplementary Fig. 4. Multi-step growth curves. The average of two separate assays is depicted in the graph. Only significant differences are depicted. Two-way ANOVA analysis was performed on log-transformed data for each time point. P-value of 0.1234(ns), 0.0332(*), 0.0021(**), 0.0002(***), <0.0001(****) are depicted accordingly.

A

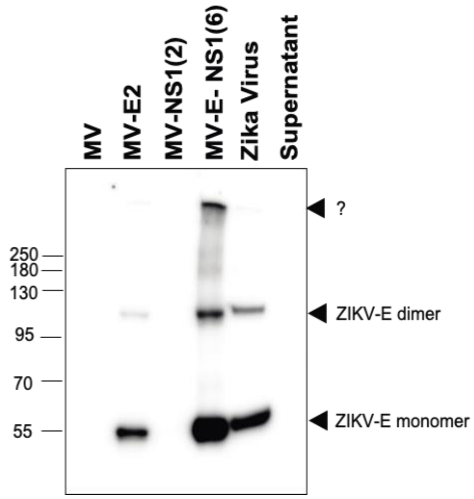

B

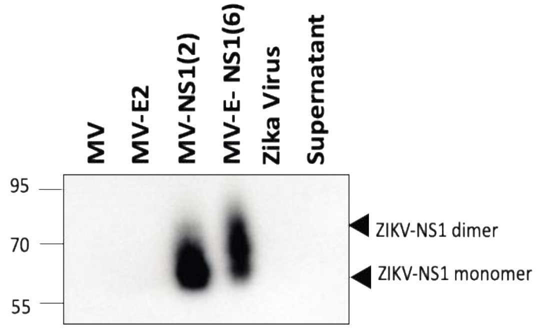

Supplementary Fig. 5

Supplementary Fig. 5: Preliminary characterization of Subviral particles.

(a) Western blot of sucrose purified SVPs probed for ZIKV-E Biofront mouse monoclonal 1176-56). **(b)** Western blot of sucrose purified SVPs probed for ZIKV-NS1 (Abcam, B4 mouse mAb) The SVP purification was performed (n=1) and assessed by western blot (n=2).

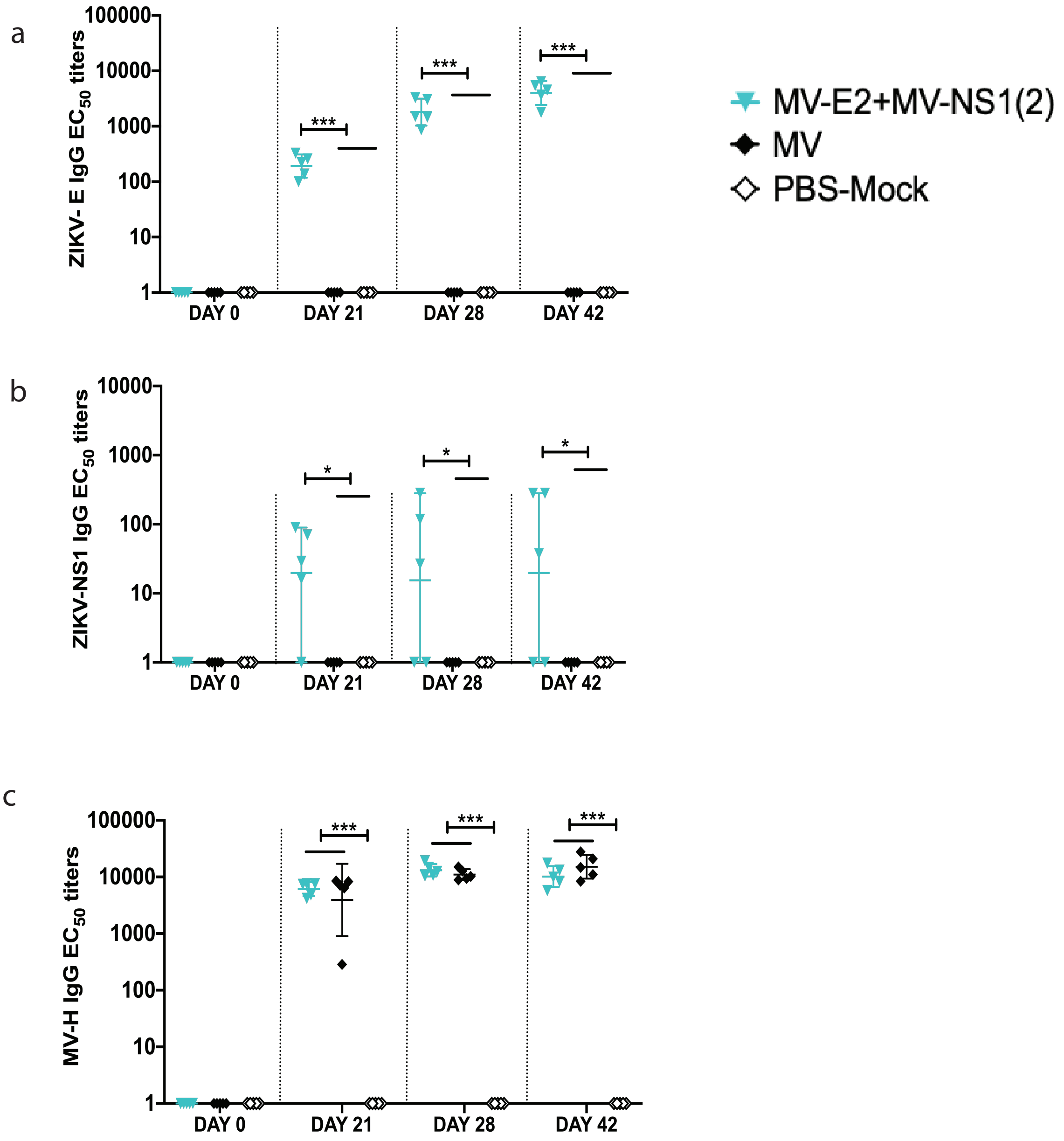

Supplementary Fig. 6

Supplementary Fig. 6. Antibody responses in the combination vaccine group

(a-c) anti-ZIKV-E (a), anti-ZIKV-NS1 (b), and anti-MV-H (c) ELISA IgG EC_{50} titers of the vaccinated animals are plotted on a graph for all animals at different time points. The mean of triplicate EC50 values is depicted per animal. The Mean ± SD is depicted per group.

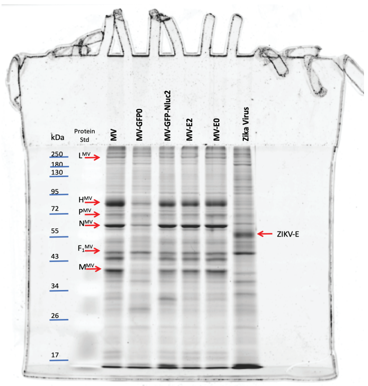

Fig. 1c

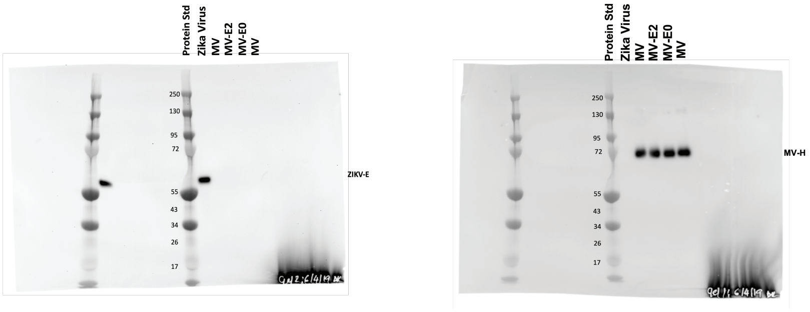

Fig. 1d

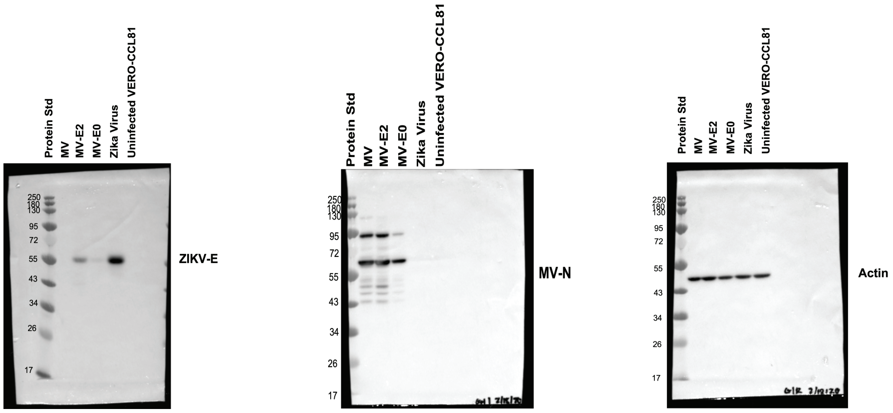

Fig. 1e

Supplementary Fig. 7

Supplementary Fig. 7. Uncropped images of Fig. 1c-e

Sypro ruby stained Protein gel (1c), Western blot of sucrose purified virions probed for ZIKV-E and MV-H (1d), Western blot of measles virus, and control virus-infected cell lysates probed for ZIKV-E, MV-N, and Actin (1e).

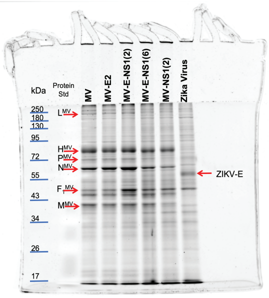

Fig. 3c

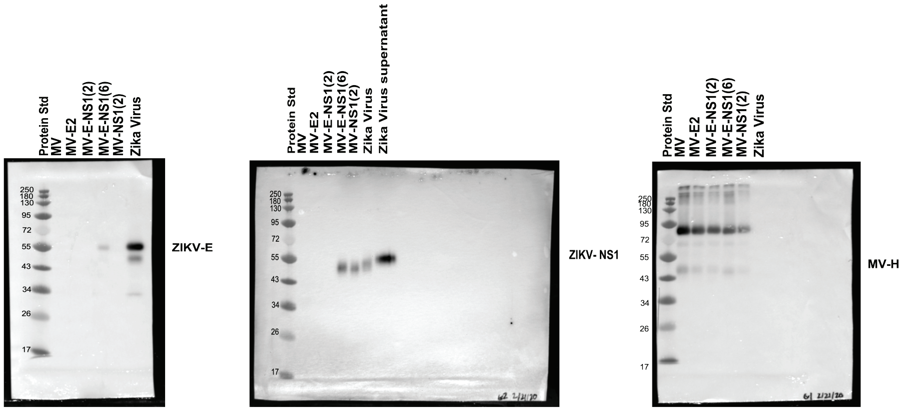

Fig. 3d

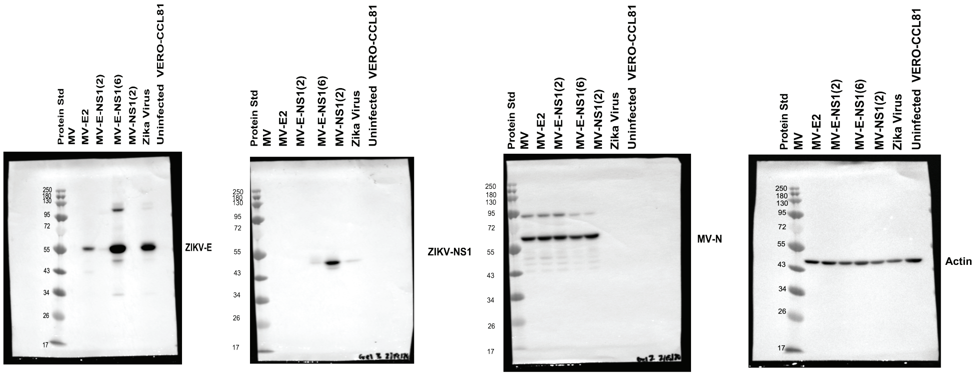

Fig. 3e

Supplementary Fig. 8

Supplementary Fig. 8. Uncropped images of Fig. 3c-e

Sypro ruby stained Protein gel (3c), Western blot of sucrose purified virions probed for ZIKV-E, ZIKV-NS1, and MV-H (3d), Western blot of measles virus, and control virus-infected cell lysates probed for ZIKV-E, ZIKV-NS1, MV-N, and Actin (3e).

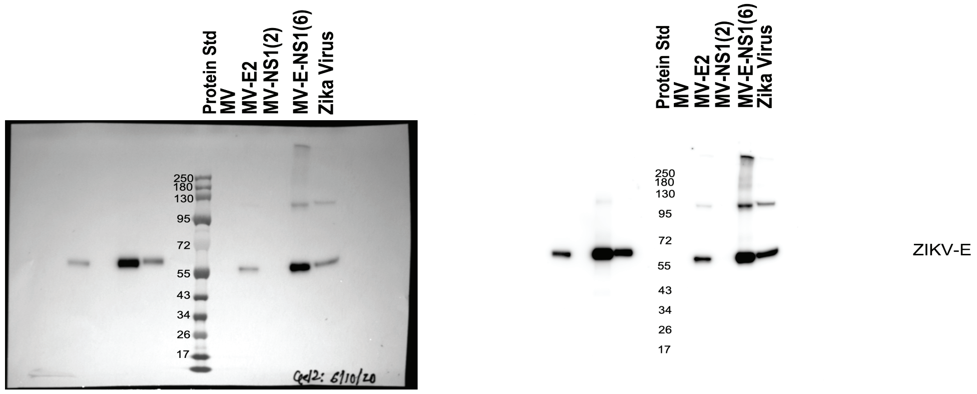

Low exposure

High exposure, without ladder (no red light)

Supplementary Fig. 5a

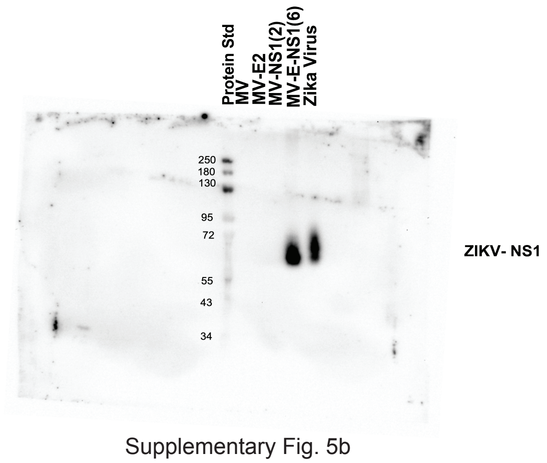

Supplementary Fig. 5b

Supplementary Fig. 9

Supplementary Fig. 9. Uncropped images of Supplementary Fig. 5a-b

Western blot of sucrose purified SVPs probed for ZIKV-E Biofront mouse monoclonal 1176-56) (5a). Western blot of sucrose purified SVPs probed for ZIKV-NS1(5b).
